# In Silico Analysis Revealed Five Novel High-Risk Single-Nucleotide Polymorphisms (rs200384291, rs201163886, rs193141883, rs201139487, and rs201723157) in *ELANE* Gene Causing Autosomal Dominant Severe Congenital Neutropenia 1 and Cyclic Hematopoiesis

**DOI:** 10.1155/2022/3356835

**Published:** 2022-05-06

**Authors:** Khyber Shinwari, Mikhail A. Bolkov, Muhammad Yasir Akbar, Liu Guojun, Svetlana S. Deryabina, Irina A. Tuzankina, Valery. A. Chereshnev

**Affiliations:** ^1^Institute of Chemical Engineering, Department of Immunochemistry, Ural Federal University, Yekaterinburg, Russia; ^2^Institute of Immunology and Physiology of the Ural Branch of the Russian Academy of Sciences, Yekaterinburg, Russia; ^3^National Center for Bioinformatics Quaid-i-Azam University Islamabad, Islamabad, Pakistan; ^4^School of Life Science and Technology, Inner Mongolia University of Science and Technology, Baotou 014010, China; ^5^Medical Center Healthcare of Mother and Child, Yekaterinburg, Russia

## Abstract

Single-nucleotide polymorphisms in the *ELANE* (Elastase, Neutrophil Expressed) gene are associated with severe congenital neutropenia, while the *ELANE* gene provides instructions for making a protein called neutrophil elastase. We identified disease susceptibility single-nucleotide polymorphisms (SNPs) in the *ELANE* gene using several computational tools. We used cutting-edge computational techniques to investigate the effects of *ELANE* mutations on the sequence and structure of the protein. Our study suggested that eight nsSNPs (rs28931611, rs57246956, rs137854448, rs193141883, rs201723157, rs201139487, rs137854451, and rs200384291) are the most deleterious in *ELANE* gene and disturb protein structure and function. The mutants F218L, R34W, G203S, R193W, and T175M have not yet been identified in patients suffering from SCN and cyclic hematopoiesis, while C71Y, P139R, C151Y, G214R, and G203C reported in our study are already associated with both of the disorders. These mutations are shown to destabilize structure and disrupt ELANE protein activation, splicing, and folding and might diminish trypsin-like serine protease efficiency. Prediction of posttranslation modifications highlighted the significance of deleterious nsSNPs because some of nsSNPs affect potential phosphorylation sites. Gene-gene interactions showed the relation of *ELANE* with other genes depicting its importance in numerous pathways and coexpressions. We identified the deleterious nsSNPs, constructed mutant protein structures, and evaluated the impact of mutation by employing molecular docking. This research sheds light on how *ELANE* failure upon mutation results in disease progression, including congenital neutropenia, and validation of these novel predicted nsSNPs is required through the wet lab.

## 1. Introduction

Neutropenia is a disease that clinicians face frequently, yet it can still be troublesome since neutrophils are the most fundamental and functionally vital component of innate immunity. Neutropenia leads to a defect in pathogen digestion, altered inflammation, an unusually severe course of infection, and a high rate of infection [1, 2].

Clinicians encounter secondary neutropenia more frequently in patients due to viral infection, autoimmune disorders, and hormonal or cytostatic therapy. Severe congenital neutropenia is a rare hematological disease characterized by a selective decrease in circulating neutrophils, bone marrow maturation arrest at the promyelocyte stage, and the occurrence of infections [3].

The causes of congenital neutropenia can be defects in neutrophil maturation and function, immune dysregulation syndromes (different familiar hemophagocytic lymphohistiocytosis), some severe combined immunodeficiencies (e.g., reticular dysgenesis (AK2 defect) and PAC2 activation defect), and primary autoimmune neutropenia at different stages of neutrophils development. Usually, patients with congenital neutropenia require antimicrobial prophylaxis and treatment with granulocyte colony-stimulating factor, and radical cure is not possible without hematopoietic stem cell transplantation [4]. There are now more than 30 inborn errors of immunity (or primary immunodeficiencies) in which neutropenia is observed, and although each condition is rare individually, the overall prevalence of these conditions in the population is serious, and a good and timely diagnosis is required to prescribe adequate therapy [5, 6].

Classical congenital neutropenia depends on the elastase function. Elastase defects lead to severe congenital neutropenia (SCN) type 1 (*ELANE* deficiency), GFI1 deficiency (SCN 2), HAX1 deficiency (SCN 3 or Kostmann disease), G6PC3 deficiency (SCN 4), VPS45 deficiency (SCN 5), type 1b glycogenosis (G6PT1 deficiency), X-linked neutropenia/myelodysplasia (WAS GOF mutation), P14/LAMTOR2 deficiency, Barth's syndrome (3-methylglutaconic aciduria, type II) (deficiency of TAZ, X-linked), Cohen's syndrome (VPS13 B deficiency), Clericuzio syndrome (USB1 deficiency), JAGN1 deficiency, 3-methylglutaconic aciduria (CLPB deficiency), G-CSF receptor deficiency (CSF3R), SMARCD2 deficiency, neutrophil specific granule deficiency (CEBPE), Shwachman-Diamond syndrome (caused by defects in at least 3 genes, SBDS, DNAJC21, and EFL1), HYOU1 deficiency, and SRP54 deficiency [4].

*ELANE* (OMIM ^#^130130) encodes neutrophil elastase, a serine protease expressed in myelomonocytic cells and their precursors. Neutrophil elastase is primarily produced during the promyelocyte stage of neutrophil maturation and is retained in the neutrophil granules of azurophils, which are involved in microorganism destruction [7, 8]. However, even when only this protein is mutated, there is a different clinical picture of congenital neutropenia, and the exact pathogenesis of each condition has not been elucidated [9–12].

The main mechanisms of neutropenia in neutrophil elastase defect are reduced to endoplasmic reticulum stress (unfolded protein response) when misfolded elastase accumulates in the endoplasmic reticulum and leads to the activation of death signals [13]. *ELANE* is known to become the most abundant protein during the developmental stage of promyelocytes, reaching millimolar concentrations in neutrophils [14], which supports the theory that accumulation of misfolded protein can cause chaperone protein deficiency, triggering death signals and apoptosis of immature neutrophils [5, 7, 8]. Another point is that mutated neutrophil elastase blocks further differentiation, leading to neutropenia [9]. The *ELANE* p. G185R polymorphism is linked with impaired neutrophil differentiation to lower expression of genes encoding critical hematopoietic transcription factors, cell surface proteins, and neutrophil granule proteins [15]. However, in all the theories of pathogenesis, the key is the degree of pathogenicity of the *ELANE* mutation, which leads to disruption of its synthesis and folding.

A single amino acid change or naturally occurring mutations are linked to a variety of complicated illnesses, including cancer. Deleterious mutations at the genomic and/or proteome levels have serious consequences for human health. Biophysics-based computational approaches are useful for examining the effects of mutations on protein structure and function, and there is a lot of interest in such research right now [16–18]. Several approaches for detecting harmful or disease-causing mutations in human protein sequences have been established. These approaches use physicochemical characteristics, structure, and cross-species conservation studies to estimate the deleteriousness of an amino acid alteration [19, 20]. The discovery of deleterious mutations in an individual has the potential to affect illness prevention as well as individualized therapies.

We used cutting-edge computational techniques to conduct an in-depth investigation of genomic and proteomic changes in *ELANE.* We looked at a variety of mutations and defined how they affect the structure and function of *ELANE* protein, which may contribute to illness onset and progression.

## 2. Methods

### 2.1. SNP Data Mining for *ELANE* Gene

*ELANE*'s FASTA sequence was obtained from UniProt (UniProt ID: P08246). A list of mutations was compiled using the dbSNP [21] and counterchecked with Ensembl [22] databases, as well as a thorough literature search at PubMed and Science Direct. During preprocessing, data redundancy, including duplicate variations, was eliminated.

### 2.2. GeneMANIA

GeneMANIA (https://genemania.org/) [23] was used to confirm the ELANE gene's linkage and examine its connection with other genes to anticipate the impact of nsSNPs on specifically related genes (accessed 10 Feb 2021 using a search strategy for ELANE in the search box). GeneMANIA uses pathways, coexpression, colocalization, genetics, protein interaction, and protein domain similarity to predict gene-gene relationships.

### 2.3. Sequence-Based Prediction and Disease Phenotype Prediction

First, the SIFT and PolyPhen2 techniques were used to determine if isolated nsSNPs are deleterious/damaging or tolerated. The SIFT method examines sequence homology and the physical characteristics of amino acid residues to assess whether a mutation is deleterious or not. It is dependent on the evolutionary conservation of amino acids in protein families. The highly conserved amino acids are often intolerant to replacements, but the less conserved ones can be replaced. A nontolerable mutation has a SIFT score of <0.05. SIFT may be accessed at https://sift.jcvi.org/ [24].

PolyPhen-2 is a tool for sequence-based mutation analysis that uses the FASTA sequence as input [25]. This program calculates the likely deleterious/damaging impacts of a mutation using conservative and physical features. It includes several sequence alignments and a machine learning-based classifier intended for high-throughput NGS data processing. It computes the mutant protein's Position-Specific Independent Count (PSIC) score and calculates the score difference with the natural protein. If the PSIC score is >0.5, the amino acid change is considered deleterious. PolyPhen-2 can be accessed at https://genetics.bwh.harvard.edu/pph2/ [26].

PROVEAN evaluates the impact of mutations on protein function based on the delta alignment score. The PROVEAN score for a deleterious mutation is <2.5, whereas scores for neutral nonsynonymous mutations are >2.5. PROVEAN Protein, PROVEAN Protein Batch, and PROVEAN Genome Variants are the three tools available on the PROVEAN online server. The SIFT tool's outputs are also delivered by the PROVEAN Protein Batch tool, which can process a higher number of protein variants. This program accepts amino acid replacements as input and works with public protein IDs such as NCBI RefSeq, UniProt, and Ensembl. PROVEAN may be found at https://provean.jcvi.org/ [27].

SNAP2 is a classifier based on a neural network. It was used to predict how a single amino acid change in the *ELANE* protein would impact its activity. This platform analyzes a FASTA sequence and assigns a prediction score (>50, strong signal for effect; −50 < score, weak signals; −100, fully neutral; +100, strong effect), reflecting the potential of a mutation affecting native protein function [28].

P-Mut is one of the platforms used to identify illness phenotypes. P-Mut is a network-based classifier that uses datasets from the manually generated Swiss-Prot database. The tool's main attributes are its physiochemical properties and conservation parts of the sequence. If a mutation has a P-Mut score > 0.5, it is considered pathogenic. The new version now includes the ability to develop new predictors for specific protein families. It also includes a database of preestimated forecasts. P-Mut may be found at https://mmb.irbbarcelona.org/PMut [29].

SNPs and GO is a website based on SVM that detects deleterious nonsynonymous substitutions [30]. It employs gene ontology (GO) annotations to determine if a missense mutation is disease-related or neutral. It accepts input in the form of an amino acid sequence/Swiss-Prot code, GO keywords, and amino acid substitutions. A disease-causing mutation is indicated by SNPs and GO scores of > 0.5, and this tool also provides PANTHER (benign is time > 450 my, probably damaging 450 my > time > 200 my, and probably benign time 200 my) and PhD-SNP results. SNPs and GO may be found at https://snps.biofold.org/snps-and-go/snpsand-go.html.

VarCARD was used to obtain findings from the MCAP (> 0.025) and FATHMM tools, FATHMM-MKL-coding-pred (> 0.5), LRT (threshold value > 0.001), METALR (> 0.5), FATHMM-pred (> 0.453), Meta-SVM, Mutation Assessor (> 0.65) (−5.545 to 5.975 (higher score > more damaging), VEST3, CAAD (> 15), DANN (> 0.5), and Mutation Taster (< 0.5). VarCARD is a genetic and medical database that includes coding variations in the human genome. A variety of genomic approaches and databases have been created to help in the study of genetic variations, particularly in nonsynonymous species. VarCARDs, on the other hand, make it simpler for scientists, researchers, general practitioners, and geneticists to collect data on a single variant or from several web platforms or databases [31].

### 2.4. Structure-Based Prediction

I-Mutant 3.0 (https://gpcr2.biocomp.unibo.it/cgi/predictors/I-Mutant3.0/I-Mutant3.0.cgi) is a predictor of the effects of single point protein mutation. The ΔΔG Mut dataset from ProTherm was used to pretrain the algorithm. A single-site mutation that depends on a protein structure or sequence can be calculated using the ΔΔG value (kcal/mol). A ΔΔG value less than zero suggests that the variant reduces protein structure or a protein sequence [32].

MUpro (https://mupro.proteomics.ics.uci.edu/) is a tool that uses machine learning vector machines and neural networks to quantify the influence of single-site amino acid modifications on protein stability. Protein stability may also be predicted using simple sequence information or a mix of sequence and tertiary structure information. The *G* cut-off value in I-Mutant3.0 and MUpro is the same [33].

### 2.5. Analysis of Conserved Residues

ConSurf is a bioinformatics instrument for precisely evaluating the rate of evolution of each position in a family of proteins. The evolutionary conservation of amino acids in a protein sequence is calculated using empirical Bayesian inference. Color palettes and conservation ratings are among the highlights. Variable amino acids were assigned a score of one, whereas the most conserved amino acid was assigned a score of nine. ConSurf was used to analyze the FASTA sequence of the *ELANE* protein and can be accessed at ConSurf.tau.ac.il [34].

### 2.6. Identification of Conserved Residues and Sequence Motifs

The human *ELANE* UniProt protein sequence was blasted at NCBI (https://blast.ncbi.nlm.nih.gov/Blast.cgi) against the UniprotKB/Swiss-Prot database, and significant alignment was identified up to 100 sequences. Clustal Omega was used to do additional computational analysis on sequences that shared more than 50% identity and had an E-value less than 1.00 E-20 [35]. The amino acid identities were colored using the Clustal color scheme, and Jalview provided the conservation index at each alignment site [36].

### 2.7. Project HOPE

Project HOPE is a web server that investigates the structural consequences of the desired mutation. The HOPE Project offers the changed protein in an observable 3D structure by cooperating with UniProt and DAS prediction algorithms. The protein sequence is used as an input source in Project HOPE, and then a structural comparison with the wild type is performed. Project HOPE is a web server that investigates the structural consequences of the desired mutation. The HOPE Project offers the changed protein in an observable 3D structure by cooperating with UniProt and DAS prediction algorithms. The protein sequence is used as an input source in Project HOPE, and then a structural comparison with the wild type is performed [37].

### 2.8. Prediction of the Amino Acid Secondary Structure Produced from the *ELANE* Gene

SOPMA [38] is a more sophisticated version of the self-optimized prediction technique (SOPM), which can predict the secondary structure (helix, turn, and coil) of 69.5 percent of amino acids in a database of 126 nonhomologous (less than 25% homologous) protein chains. SOPMA and a neural network approach (PhD) properly predict 82.2 percent of residues and 74% of copredicted amino acids when used together.

### 2.9. Protein Domain Identification and Confirming the Presence of Deleterious nsSNPs in Domains

SMART (Simple Modular Architecture Research Tool) is a tool for identifying and annotating genetically mobile domains as well as analyzing domain structures (https://SMART.embl-heidelberg.de). Searches for proteins containing specified combinations of domains in defined taxa are possible using the database's user interfaces. We utilized a protein sequence in FASTA format or a protein ID as a query to predict domains and motifs [39].

### 2.10. Ligand-Binding Sites Predictions

RaptorX binding (https://raptorx.uchicago.edu/BindingSite/) is a web portal that predicts the binding sites of a protein sequence using a RaptorX-predicted 3D model [40]. RaptorX predicts secondary and tertiary protein structures, in addition to disordered areas and distance maps, solvent accessibility, and binding sites. One factor of pocket multiplicity is employed in the prediction of binding sites concerning *P*-value, uGDT (GDT), and uSeqID (SeqID). Higher values suggest that the expected pocket is more reliable, especially when the score is close to 40.

### 2.11. PTM Sites Prediction in ELANE Protein

Posttranslational modifications (PTM) are used to predict the function of a protein. [41] GPSMSP v3.0 (https://msp.biocuckoo.org/online.php) was utilized to predict methylation sites in the *ELANE* protein. We used NetPhos 3.177 (https://www.cbs.dtu.dk/services/NetPhos/) [42] and GPS 5.078 (https://gps.biocuckoo.cn/) [43] to predict potential phosphorylation sites. The NetPhos 3.1 service predicts serine, threonine, and tyrosine phosphorylation sites in proteins using neural network ensembles. Phosphorylation is indicated by residues in the protein with a score greater than 0.5. A higher GPS 5.0 score, on the other hand, suggests that you are more likely to be phosphorylated. To assess potential methylation and ubiquitylation sites, we utilized GPSMSP 1.0 (https://msp.biocuckoo.org/), BDMPUB (https://www.bdmpub.biocuckoo.org), and UbPred [44] (https://www.ubpred.org). NetOglyc4.0 additionally predicts glycosylation sites using glycosylation [45] (for further information, visit https://www.cbs.dtu.dk/services/NetOGlyc/). Sites of glycosylation with a score greater than 0.5 are more likely to be glycosylated.

### 2.12. Predicting 3D Protein Structure

Phyre2 is a 3D homology modeling tool that predicts 3D models for proteins (http://www.sbg.bio.ic.ac.uk/phyre2/html/page.cgi?xml:id=index) [46]. The researchers constructed 3D models of the wild type and 22 mutants linked to the most harmful nsSNPs. The researchers compared wild-type ELANE and selected alterations using TMalign (https://zhanglab.ccmb.med.umich.du/TM-align/) [47]. The template modeling score (TM-score), root mean square deviation (RMSD), and structural superposition are all predicted. The TM scores vary between 0 and 1, with a higher value indicating greater structural similarity. The RMSD increases as the difference between mutant and wild-type structures grow. Three mutants with lower RMSD values were submitted to the ITASSER (https://zhanglab.ccmb.med.umich.edu/I-TASSER%20R/) for 3D protein structure comparisons [48–50]. Chimera v1.11 was used to analyze molecular characteristics and interactively show the final protein structure [51]. PROCHECK [52] was used to validate the 3D models.

### 2.13. Molecular Docking Analysis of *ELANE*

The graphical user interface tool AutoDock was used to perform intermediate procedures such as producing pdbqt files for protein and ligand preparation and generating grid boxes (ADT). ADT was used to determine the protein polar hydrogens, Kollman charges, solvation parameters, and fragmental volumes. The produced file was saved in PDBQT format by AutoDock. AutoGrid and a grid box were used to produce a grid map. The grid was configured to 40 × 40 × 40 xyz points with a grid spacing of 0.375 with grid centers of −49.009, 14.545, and 34.19 (*x*, *y*, and *z*). The ligand structure is employed to build a scoring grid, which shortens the computation time. AutoDock/Vina was used to bind proteins and ligands using protein and ligand information. AutoDock/Vina uses an iterated local search global optimizer, and during docking, both the protein and the ligands are treated as stiff [53].

### 2.14. Statistical Analysis

Correlation analysis of the predictions generated by computational in silico technologies was conducted using SPSS v23 and MS Excel. Student's *t*-test was performed to examine the significant differences predicted by the various computational techniques. A *P*-value of less than 0.01 was judged statistically significant.

## 3. Results

### 3.1. Exploring the Desired Gene Using dbSNPs/NCBI

**Figure 1:**
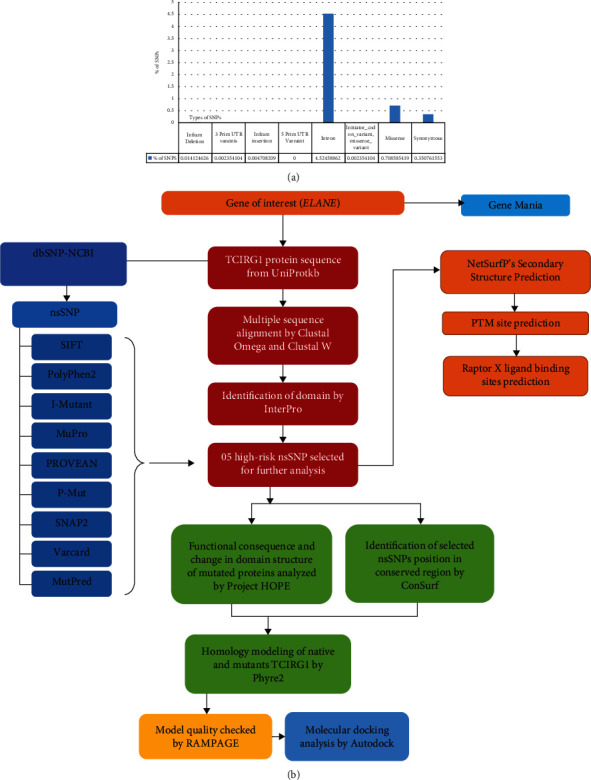
(a) Number of mutations reported in ELANE, extracted from the dbSNP database. (b) Flowchart for methodology.

SNPs in the ELANE gene were reported using the NCBI database (https://www.ncbi.nlm.nih.gov/). It consists of 3646 SNPs, 301 of which are in coding nonsynonymous areas (missense) and 149 of which are in synonymous parts, all of which were present in *Homo sapiens* (Figure 1(a)). The schematic of the research methodology is given in Figure 1(b).

### 3.2. GeneMANIA

**Figure 2:**
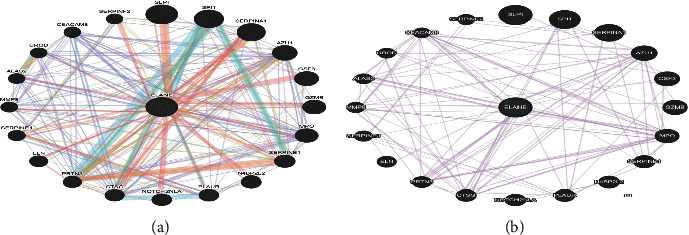
(a) Gene-gene interaction of *ELANE* with other genes proposed by GeneMANIA. (b) Coexpression in GeneMANIA.

The ELANE gene encodes a protein present in the extracellular matrix of proteins and other substances. Our findings revealed that ELANE is co-expressed with 20 genes (SERPINA1, AZU1, GZMB, CTSG, PRTN3, SERPINB1, SERPINE1, SERPINF2), shared domain with only 8 genes (SERPINA1, AZU1, GZMB, CTSG, PRTN3, SERPINB1, SERPINE1, SERPINF2), physical interaction with 19 genes (SERPINA1, AZU1, GZMB, CTSG, PRTN3, SERPINB1, SERPINE1, SERPINF2), and co-localization with 19 genes (ARSA, TYMP) (Figure 2).

### 3.3. SIFT and PolyPhen Predictions of ELANE Gene nsSNPs

A total of 301 single-nucleotide polymorphisms (nsSNPs) were studied to see if they influenced protein structure or function. The first step was to determine which of the nsSNPs is responsible for the amino acid substitution. SIFT computes the effect of an nsSNP on protein structure and determines if the induced amino acid is acceptable at that location. SIFT predicted 21 nsSNPs to be deleterious, while 18 nsSNPs were predicted to be deleterious together by SIFT and PolyPhen out of a total of 301 nsSNPs (Table 1 and Supplementary Table S1.1).

### 3.4. The Most Deleterious SNPs Identified in ELANE

Both the SIFT and PolyPhen algorithms offer 18 common pathogenic nonsynonymous SNPs. To confirm the pathogenicity of the identified mutations, we analyzed the pathogenicity of nsSNPs through other tools. However, not every platform produces similar results.

For example, the FATHMM-MKL algorithm also determined all nsSNPs detected by SIFT and PolyPhen as pathogenic (Table 2). However, all other algorithms used in this work did not show a 100% match, as did SIFT, PolyPhen, and FATHMM-MKL.

Among all the 21 SIFT nsSNPs predicted, the LRT and FATMANH algorithms predicted the least number of matching results. Both algorithms predicted only 10 pathogenic nsSNPs for *ELANE* and 11 as tolerant, neutral, or unknown.

The PolyPhen platform identified 18 deleterious nsSNPs; the VEST, CADD, and DANN platforms each predicted 19; MCAP and Mutation Taster each predicted 20 deleterious nsSNPs. Using the SNAP2 approach, 18 mutations were found to be damaging, whereas three had no association with pathology.

**Figure 3:**
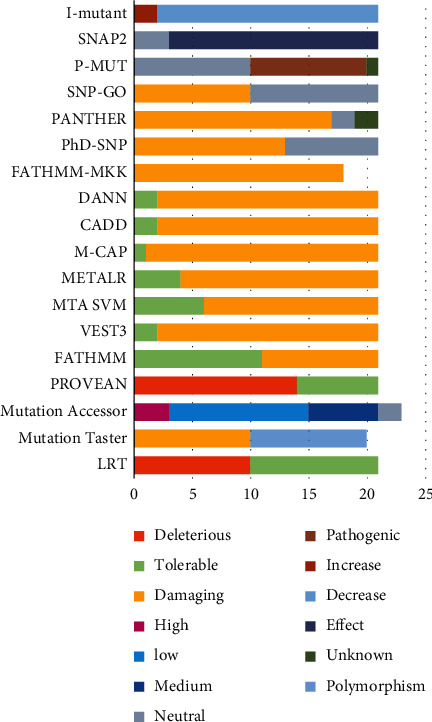
Prediction results of the 21 SIFT and PolyPhen2 deleterious nsSNPs in the *ELANE* gene analyzed by the eighteen computational tools.

For the PANTHER program, 17 nsSNPs were considered deleterious mutations, among which 10 nsSNPs were classified as probably pathogenic, 7 classified as possibly pathogenic, 2 marked as probably benign, and 2 variants with unknown significance (Figure 3 and S2). When analyzed with PROVEAN, 14 of the 21 nsSNPs in the *ELANE* gene were predicted to be highly disruptive, and 7 were considered neutral.

Mutation Assessor counted 20 nsSNPs as destructive, including 3 with high pathogenicity, 6 with medium, 12 with low, and 1 with an unknown value. P-Mut predicted 10 mutations as pathological, 10 with unknown significance, and 1 with no result. PhD-SNP predicted 13 mutations as pathological, SNP-GO 10, METALR 17, and MTA-SVM 15.

All current nsSNP pathogenicity assessment methods used together identified 8 overlapping common mutations in the *ELANE* gene: C71R, P139L, C151Y, T175M, G203S, G214R, R193W, and F218L.

According to the software used, we know that the frequency of the C71R allele in Latinos is 3.655e-05 and T175M in Africans is 0.0002, in Latinos is 0.0023, in East Asians is 5.832e-05, in Nephinese Europeans is 3.632e-05, and in Latinos is 2.979e-05. The results of all prediction algorithms were statistically significant and closely related. The value of the Student coefficient between the instruments has a *P*-value of 0.001.

In addition to SIFT and PolyPhen2, the results of pathogenic mutation prediction by the above tools as well as the predictive significance of all tools are presented in Figure 3 (Supplementary Table S1.1 and Supplementary Figure S2.1).

### 3.5. Identification of Domains in *ELANE* Gene

**Figure 4:**
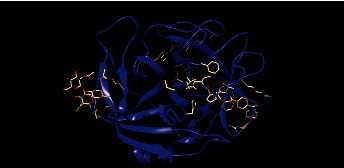
Identification of nsSNPs in the domains of *ELANE* gene protein.

The SMART online database was used to identify domains in the *ELANE* gene and nsSNPs in different domains. SMART may look for proteins that have the same domain architecture as the query (i.e., all of the domains in the query protein are in the same collinear order) or the same domain composition as the query (at least one of all domain types of the query protein, irrespective of order). Proteins with similar or identical domain structures to the query protein may improve predictions of protein activities rather than domain functionalities. This database yielded the domain trypsin-like serine protease (29–242), with an *E*-value of 1.78444685325842e-71 for *ELANE* protein neutrophil elastase. Mutations in this domain induce congenital neutropenia, and one of our most dangerous nsSNPs is situated in this domain.  Figure 4 depicts the three-dimensional structure of neutrophil elastase, which contains a trypsin-like serine protease domain.

### 3.6. Prediction of Stability of the Mutated Protein due to SNPs by I-Mutant 3.0 and MUpro

The online software I-Mutant 3.0 was used to forecast the consequences of *ELANE* high-risk nsSNPs on protein stability and function. The results revealed that V101L and A166V had enhanced protein stability, whereas R34W, C71R, V101M, P139L, R143C, C151Y, A166T, T175M, R182H, V190M, R193W, G203S, L206F, N209K, G210R, G214R, F218L, P262S, and P262L had decreased protein stability. At the same time, MUpro showed all deleterious nsSNPs decrease the protein stability. I-Mutant 3.0 and MUpro results are provided in Supplementary Table S3.1.

### 3.7. Association of SNPs with Highly Conserved Buried (Structural) and Exposed (Functional) Amino Acid Residues in ELANE Protein

**Figure 5:**
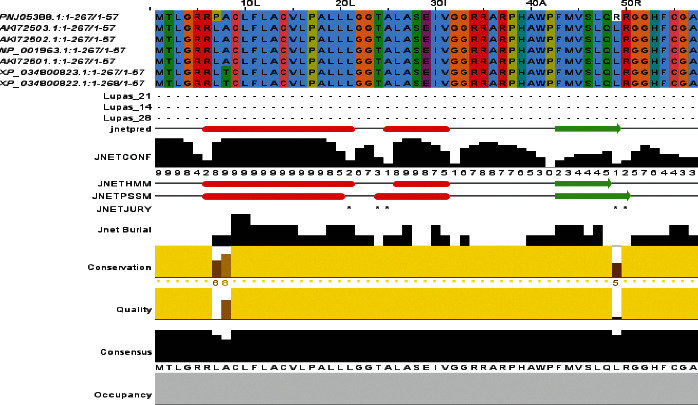
Amino acid alignment of human *ELANE* (UniProt ID: P08246) along with its homologues in phylogenetically close species in AK172501.1, XP 034800823.1, and XP 034800822.1. Solid horizontal bars indicate conserved sequence motifs, and residues with asterisk (^*∗*^) mark indicate evolutionarily conserved amino acids. The amino acid identities were colored according to the Clustal color scheme, and the conservation index at each alignment position was provided by Jalview.

The *ELANE* gene regulates the synthesis of neutrophil elastase, a protein. This protein is present in neutrophils, which are white blood cells that help fight infection and inflammation. Neutrophils secrete neutrophil elastase as the body develops an immunological response to combat an infection. To resist infection, this protein alters the activity of specific cells and proteins. The *ELANE* protein sequence was aligned using Clustal Omega with default parameters after blasting against UniprotKB/Swiss-Prot entries. TC protein sequences aligned with other phylogenetically related sequences from other living organisms, such as NP 001963.1 (*Homo sapiens*), AK172501.1 (synthetic construct), AK172502.1 (synthetic construct), AK172502.3 (synthetic construct), XP 034800823.1 (*Pan paniscus*), XP 034800822.1 (*Pan paniscus*), and PNJ05388.1 (Figure 5). The results give a colorimetric conservation score ranging from 1 to 10. There are twenty-one conserved residues in all.

### 3.8. Conservation Analysis

**Figure 6:**
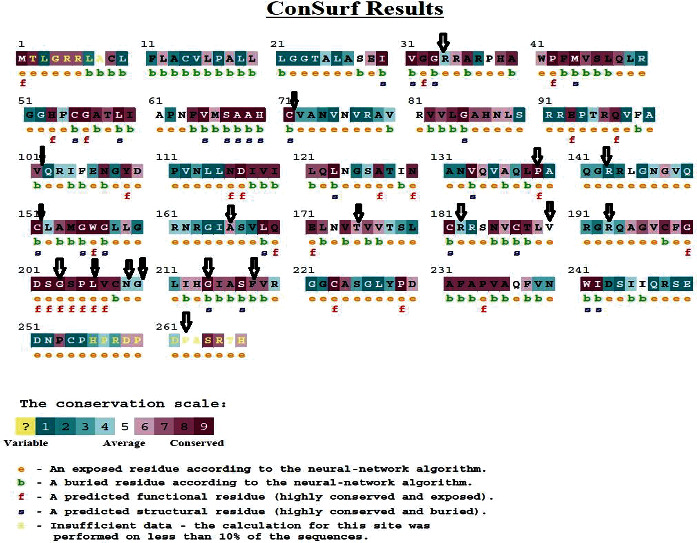
Sequence conservation analysis of the *ELANE* protein using ConSurf web server.

We investigated the degree of conservation of *ELANE* residues using the ConSurf web server. According to the results of the ConSurf investigation, 22 dangerous missense SNPs are found in highly conserved areas (7-8-9). Among these 22 missenses variants, 8 were located in the highly conserved positions, 2 (P139L and C71R) were predicted as functional and exposed residues, and the other 3 (G214R, C151Y, and C71Y) were predicted as buried and structural residues. Twelve (R34W, R143C, A166T, A166V, T175M, R182H, V190M, R193W, N209K, G210R, P262S, and P262L) were predicted as exposed and the other 3 (F218L, V101 L, and V101 M) were buried residues. The results are shown in Figure 6.

### 3.9. Project HOPE

Because all identified *ELANE* nsSNPs were considered high-risk mutations by prediction, the HOPE service was used to predict their impact. The HOPE was based on amino acids' size, spatial arrangement, charge, hydrophobicity, structure, and function. One mutant amino acid was smaller than its wild-type equivalent, while seventeen mutant amino acids were bigger. At three different places, the charge was altered from positive to neutral, from neutral to positive at four spots, and from neutral to negative at one location. The hydrophobicity of eight of the alterations increased, while the hydrophobicity of the other remained the same. This study suggests that amino acid modifications at these sites alter protein structure and interactions with other molecules, hence affecting protein function. The results are shown in the following graph (Supplementary Table S4.1).

### 3.10. Prediction of Amino Acid Secondary Structure of the Protein Corresponding to *ELANE*

**Figure 7:**
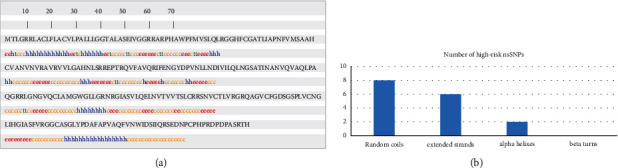
(a) SOPMA predicted distribution of high-risk *ELANE* nsSNPs in random coils, alpha-helices, extended strand, and beta-turns. (b) SOPMA study of the secondary structure of individual amino acid residues in *ELANE* gene-derived protein. The high-risk nsSNPs are indicated by the boxes.

SOPMA predicted the secondary structure of *ELANE*, which explained the alpha-helix, beta-sheet, and coil distributions. In the projected secondary structure, there were a lot of random coils (128, 47.94 percent), followed by 70 extended strands (26.22 percent), 56 alpha-helices (20.97 percent), and 13 beta-turns (4.87 percent) (Figure 7(a)). For the 21 amino acid residues that correspond to 16 high-risk nsSNPs, 8 were located in random coils, 2 in alpha-helices, 6 in extended strands, and 0 in beta-turn (Figure 7(b)).

### 3.11. Predicted PTMs (Posttranslation Modifications)

GPSMSP 3.0 was utilized for this purpose, and it projected that no *ELANE* sites would be affected. *ELANE* phosphorylation sites predicted by GPS 3.0 and NetPhos 3.1 are listed in Supplementary Table 5.1. NetPhos 3.1 projected that 62 residues (Ser 09, Thr 03, and Tyr 01) had phosphorylation potential. GPS 3.0, on the other hand, did not provide a phosphorylated result. For ubiquitylation prediction, BDMPUB and UbPred were utilized. UbPred anticipated that none of the lysine residues would be ubiquitinated, but BDMPUB predicted that 11 lysine residues would be ubiquitinated. None of the BDMPUB predictions were located in a highly conserved or detrimental nsSNP region. NetOGlyc4.0 was used to predict potential glycosylation sites. None of those predicted by BDMPUB were found in a highly conserved or deleterious nsSNP area. NetOGlyc4.0 was used to forecast probable glycosylation sites. The results indicated that all potential glycosylation sites in 25 locations were predicted to be glycosylated with varying scores in wild-type *ELANE* protein. Interestingly, the mutant *ELANE* sequence does not differ from the natural in terms of glycosylation sites. All the scores for the wild‐type and mutants are given in Supplementary Table S5.2 and Supplementary Table S5.3.

### 3.12. Ligand-Binding Site Prediction by RaptorX

When using the RaptorX binding server, the criterion for accurate prediction is a pocket multiplicity number greater than 40. The *ELANE* protein had the highest pocket multiplicity of 71, which were linked to the residues H70, L114, G196, V197, C198, F199, G200, S202, A216, S217, F218, V219, G222, C223, and D230 with an expected SO4, BEN, and ANH ligand. The *ELANE* protein had the 2nd highest pocket multiplicity of 46, which were linked to the residues N88, R91, E93, and R96 with ligand CA.

### 3.13. 3D Modeling of *ELANE* and Its Mutants

**Figure 8:**
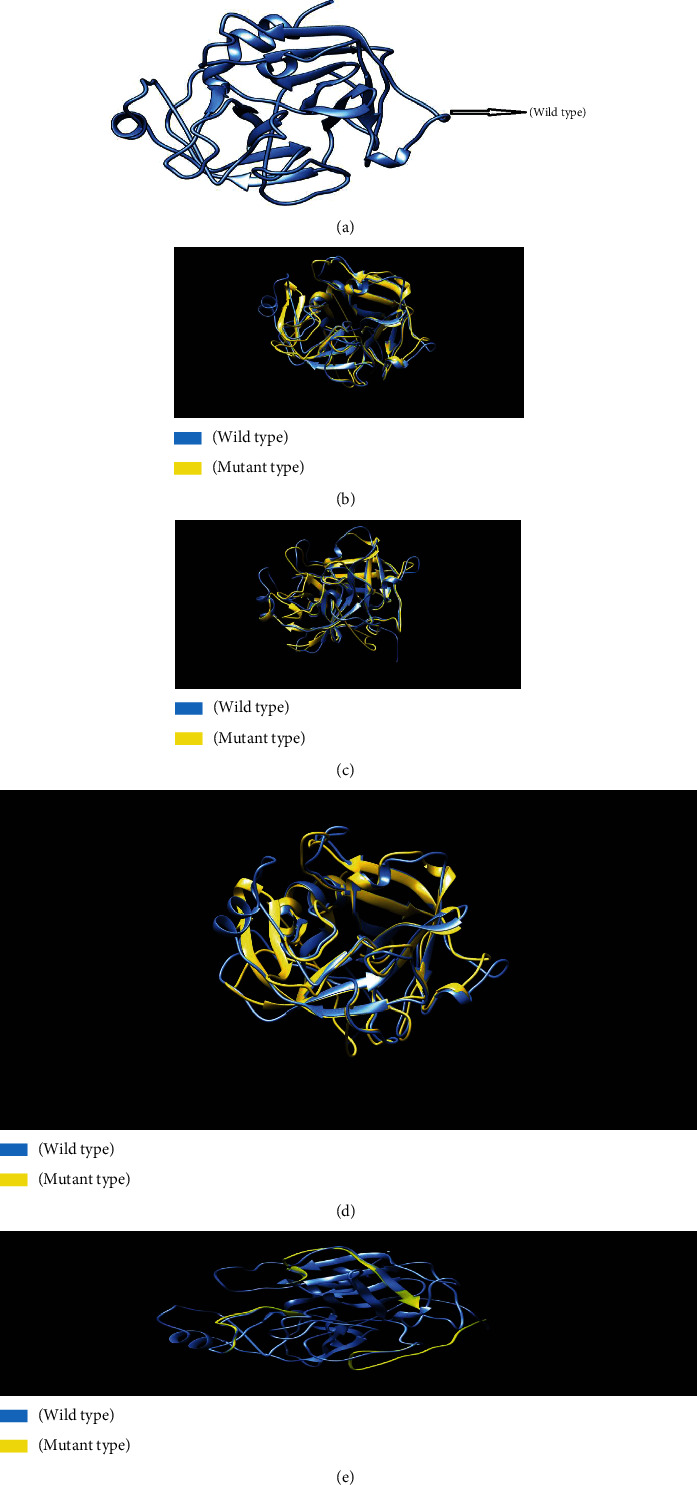
(a) Wild‐type *ELANE* protein structure. (b) Superimposed structure of ELANE and its C71R mutant. (c) Superimposed structure of *ELANE* and its F218L mutant. (d) Superimposed structure of ELANE and its R34W mutant. (e) Superimposed structure of *ELANE* and its G214R mutant.

Phyre2 was used to model 3D structures of the wild-type *ELANE* protein as well as four very harmful nsSNP mutations. Separate nsSNP substitutions in the *ELANE* protein sequence were produced and then submitted to Phyre2 [46] which predicted the 3D structures of the mutant proteins. Phyre2 picked c6o1gA as a template for 3D model prediction because it had the highest resemblance, according to the Phyre2 server, which can be seen in Figure 8. TM scores and RMSD values were calculated for each mutant model. The TM-score represents topological similarity, whereas the RMSD values indicate the average distance between the carbon backbones of wild and mutant models. Higher RMSD values indicate that the mutant structure deviates from the wild type. The mutant C71Y (rs28931611) model had the highest deviation with a 2.05B RMSD value, followed by R34W (rs201163886), F218L (rs200384291), and G214R (rs137854451) with 1.98B, 1.96B, and 1.12B RMSD values, respectively. P139L, G203S, and R193W have RMSD values of 0.04B, 0.49B, and 0.96B, respectively, indicating no structural difference from wild type.  Table 3 shows the scores and RMSD values. Four nsSNPs with the greatest RMSD values (C191Y, G214R, R34W, and F218L) were chosen and submitted to ITASSER for remodeling. The protein structure produced by the ITASSER is the most dependable since it is the most powerful modeling tool.  Figure 7 depicts the three mutations overlaid on the wild-type *ELANE* protein using Chimera 1.11. The validation findings for the wild and mutant versions of the 3D models were satisfactory, and the Ramachandran plots and Chi1-chi2 plots of the most detrimental nsSNPs can be seen in Supplementary File 6 (Supplementary Figure S6.1).

### 3.14. Docking Analysis

**Figure 9:**
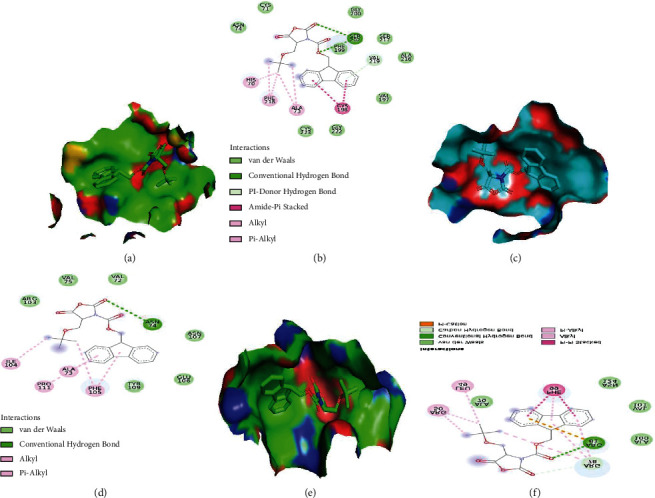
2D and 3D surface plots with ligand inside the active pocket. (a, b) Wild type. (c, d) Mutant C71Y. (e, f) Mutant F218L.

The docking conformations of all hits were visually evaluated using Discovery Studio and PyMol, and their interactions were calculated to discover the binding forces that were crucial in stabilizing the formation of receptor-ligand complexes. The ligand ANH was docked into the active site of the wild-type protein as well as four mutant proteins. The docking score for the wild type was −8.4 KJ/mol, and the 2D interaction showed that the wild type has two hydrogen bonds with SER202, as well as seven van der Waals and seven hydrophobic contacts (Figures 9(a) and 9(b)). The docking scores for the mutations G214R, R34W, C71Y, and F218L were −9.2, −7.5, −7.1, and −6.8 KJ/mol, respectively. The 2D interaction of G214R shows 2 hydrogen bonds with SER202, 7 van der Waals, and 5 hydrophobic interactions. R34W shows 2 hydrogen bonds with SER202 and VAL219, 6 van der Waals interactions, and 8 hydrophobic interactions. One hydrogen bond is present in mutation C71Y with ARG81, 4 van der Waals, and 8 hydrophobic interactions. Similarly, F218L shows 1 hydrogen bond with ASN74, 6 van der Waals, and 4 hydrophobic interactions (Figures 9(c)–9(f)). Two mutations, G214R and R34W, have interactions that are quite close to the wild type. All of them are engaged in hydrogen bonding with SER202. The other two mutations (C71Y and F218L) have fewer hydrogen bonds, indicating that these two mutations may influence protein stability and energy. Figures 9(c)–9(f) depict the surface depiction of the active site of the protein with the ligand.

## 4. Discussion

This work uses a comprehensive computational method based on diverse biophysical techniques to investigate the impact of mutations on *ELANE* structure and function. However, there are far too many nsSNPs in *ELANE* that might be candidate disease indicators and have a role in the illnesses produced by this gene. In this study, the dbSNP database revealed 301 missense nsSNPs in the *ELANE* gene, with 5 of the known MAFs of nsSNPs in the *ELANE* gene being less than 1%. SIFT predicted 21 nsSNPs as deleterious, while PolyPhen overlapped with SIFT 18 nsSNPs in the *ELANE* gene as potentially deleterious. All of the state-of-the-art prediction approaches used in the study confirmed the top eight high-risk nsSNPs (C71R, P139L, C151Y, T175M, G203S, G214R, R193W, and F218) in (Table 2) as exceedingly deleterious/damaging. The mutants F218L, R34W, G203S, R193W, and T175M have not yet been identified in patients suffering from SCN and cyclic hematopoiesis, while C71Y, P139R, C151Y, G214R, and G203C reported in our study are already associated with both of the disorders. R34W deleterious nature was not confirmed by all of the prediction tools; further wet lab/dry lab investigations are required. These five nsSNPs can work as markers to identify additional people with *ELANE* gene disorder.

There were few nsSNPs (R143C, A166V, R182H, V190M, L206F, N209K, and G210R) that were novel but they were not deleterious by all of the in silico tools used in the study. These SNPs will need further confirmation to consider in a study for patients. ConSurf assesses if an amino acid is conserved, exposed, functional, or structured using a mix of evolutionary conservation data and solvent accessibility predictions. Based on their placements on the protein surface and core, highly conserved residues are considered to be structurally or functionally significant [34]. Amino acids, which are involved in protein-protein interactions, should be more conserved. The most damaging nsSNPs are those that are present in conserved regions [54]. In a total of 22 nsSNPs, only 8 (C71Y, P139L, C151Y, T175M, G203S, G214R, R193W, and F218L) are present at evolutionary conserved, exposed, and functionally important residues. There were 12 nsSNPs (R34W, R143C, A166T, A166V, T175M, R182H, V190M, R193W, N209K, G210R, P262S, and P262L) located at conserved, buried, and structurally important residues. The remaining nsSNPs were located in either exposed or buried residues, and none of them had any structural or functional effect on the *ELANE* protein.

Protein stability was predicted using the I-Mutant and MUpro web server, and variants R34W, C71R, V101M, P139L, R143C, C151Y, A166T, T175M, R182H, V190M, R193W, G203S, L206F, N209K, G210R, G214R, and F218L were predicted to decrease the stability of the protein. These nsSNPs in the future will be important during the study of the *ELANE* gene since they reduce protein stability. Various in silico investigations on genes and proteins such as *ADA* and *GJA3* have been conducted [55, 56].

This type of study might lead to the discovery of novel treatment. RAMPAGE data were used to validate all of the simulated structures. For the structure given in Figure 5(a) (*ELANE* wild type), RAMPAGE values were 82.3% favored residues, 15.6% allowed, 2.2% generally allowed, and 0.0% disallowed, similarly for mutants C71Y (84.2% favored residues, 12.6% allowed, 2.1% generally allowed, and disallowed 1.1%), R34W (84.6% favored residues, 12.2% allowed, 1.6% generally allowed, and disallowed 1.6%), F218L (83.6% favored residues, 12.7% allowed, 2.6% generally allowed, and disallowed 1.1%), and G214R (81.7% favored residues, 15.7% allowed, 2.6% generally allowed, and disallowed 0.0%). Secondary structures include the alpha-helix, beta-sheet, and coil. PTMs are important in cell signaling, protein-protein interactions, and other processes in biological systems [57, 58]. They impact protein shapes and functions. We wanted to examine if the chosen nsSNPs modified the PTMs of the *ELANE* protein. Several bioinformatics approaches were used to predict PTM sites in the protein under study. Methylation is a significant PTM because lysine residues in proteins are methylated, influencing their interaction with DNA and gene expression. The molecular switch is another protein regulatory mechanism that adjusts the protein to perform such as protein structure conformational changes, protein activation and deactivation, and signal transduction pathways [59–62].

According to the ConSurf Conservation profile, several predictions are highly preserved, exposed, and functionally relevant, highlighting their importance. Ubiquitylation is a protein degradation mechanism that helps DNA damage repair [63]. We performed molecular docking research to see if the C71Y and F218L mutations had a deleterious effect on the *ELANE* protein. Docking experiments revealed that both mutations drastically damaged *ELANE*'s binding pocket with ATP. Normal protein was compared to mutant, and the most evident difference was found in F218L, showing a significant reduction in H-bond interactions inside the binding pocket residues. Because ATP is tightly bound to *ELANE*'s binding cleft, these mutations interfere with the favorable interactions essential for *ELANE*'s functional activity. Furthermore, the difference in the bound ATP molecule may reduce *ELANE*'s catalytic efficiency. It is essential for protein function and stability. It serves as a structural component in protein-protein interactions. PTM predictions show that phosphorylation is the sole PTM that may have a significant influence on *ELANE* protein structure and function, with the Supplementary File S4 containing the most critical phosphorylation sites.

According to GeneMANIA's predictions, *ELANE* is the most interacting gene and coexpressed with diverse genes in our study. Because of their interaction patterns and coexpression profiles, any of the most deleterious nsSNPs in the *ELANE* gene will ultimately influence and impair the proper functioning of other linked genes. This emphasizes the importance of these interconnected and coexpressed genes in congenital neutropenia and other primary immunodeficiency diseases. Because our study was comprehensive, it included all of the necessary data and analyzes for identifying the most harmful nsSNPs. Every study, including ours, had limitations.

Our study is based on mathematical and statistical approaches used in computer tools and web servers. As a result, further investigation will be beneficial to validate these findings. Our findings provide information on the *ELANE* gene's deleterious nsSNPs, protein 3D structure, PTM potential sites, and gene-gene interaction with other genes, all of which might be relevant in the future while studying the *ELANE* gene and its disorders. Raptor*X* predicted 15 ligand-binding sites, each of which is H70, L114, G196, V197, C198, F199, G200, S202, A216, S217, F218, V219, G222, C223, and D230 residues. As a result, the highly toxic F218L is identified in the ligand-binding domain and forms the catalytic coordination sphere, potentially altering the *ELANE* protein's binding affinity.

## 5. Conclusion

Single amino acid changes are among the most common mutation variants associated with various disorders, including neutropenia. The wide variety of protein variants suggests different degrees of functionality and, in certain situations, determines the direction of pathology or disease resistance. An in-depth study of amino acid substitutions will correlate protein features and clinical conditions, develop effective therapeutic agents, and predict the course of the disease for the patient. This study describes five (R34W, T175M, G203S, R193W, and F218) nsSNPs that are lethal and have mutational effects on *ELANE* protein structure and function, and these mutations can affect wild-type protein structure. These mutant structures exhibit significant differences in complex binding, which may impair *ELANE* protein activation and reduce the efficiency of trypsin-like serine protease. These differences could potentially compromise the validation of the secondary structure, threatening the stability of the protein. We also observed that the ability of the mutant proteins to bind ATP was lower than that of the wild-type protein. However, these five nsSNPs mutations have not yet been described in humans with autosomal dominant severe congenital neutropenia *ELANE* 1. As a result, these newly uncharacterized nsSNPs may cause disease by affecting protein activation or efficiency. The results of this study will help future genomic association studies to identify deleterious SNPs associated with different forms of autosomal dominant severe congenital neutropenia 1. Large-scale clinical trial-based studies, as well as experimental mutation studies, are needed to characterize these data on SNPs.

## Figures and Tables

**Table 1:** SIFT and PolyPhen deleterious nsSNPs.

ID of nsSNPs	AA position	SIFT	Score	PolyPhen	Score	Allelic frequency
rs201163886	R34W	Deleterious	0.002	Probably damaging	1	
rs28931611	C71R	Deleterious	0	Probably damaging	1	6.076e-06
rs137854449	V101M	Deleterious	0.005	Probably damaging	0.964	
rs137854448	P139L	Deleterious	0	Probably damaging	1	
rs199558534	R143C	Deleterious	0.048	Probably damaging	1	
rs57246956	C151Y	Deleterious	0	Probably damaging	1	
rs201788817	A166T	Deleterious	0.33	Probably damaging	0.976	
rs199891906	A166V	Deleterious	0.23	Probably damaging	0.582	
rs193141883	T175M	Deleterious	0.008	Probably damaging	1	gnomAD_exome 0.0005
rs200449787	R182H	Deleterious	0.015	Probably damaging	1	
rs367663236	V190M	Deleterious	0.047	Probably damaging	1	
rs201723157	R193W	Deleterious	0.006	Probably damaging	1	
rs201139487	G203S	Deleterious	0	Probably damaging	1	4.094e-06
rs137854446	L206F	Deleterious	0	Probably damaging	1	
rs201664319	N209K	Deleterious	0.03	Probably damaging	0.983	
rs140880838	G210R	Deleterious	0.019	Probably damaging	1	
rs137854451	G214R	Deleterious	0.002	Probably damaging	1	
rs200384291	F218L	Deleterious	0.011	Probably damaging	0.998	

Threshold: SIFT: < 0.05, PolyPhen2: > 0.8 (PSIC > 0.5), or benign (PSIC < 0.5).

**Table 2:** Prediction of highly pathogenic nsSNPs predicted to be deleterious by all the prediction tools.

AAS	LRT	Mutation Taster	Mutation Assessor	PROVEAN	FATHMM	VEST3	MTA-SVM	METALR	MCAP	CADD	DANN	FATHMM-MKK	PhD-SNP	PANTHER	SNP-GO	P-MUT	SNAP2
C71Y	0.001 (U)	1 D	4.21 H	−11.22 D	−4.7 D	0.944 D	1.097 D	0.968 D	0.973 D	23.9 D	0.987 D	0.817 D	0.933 D	0.907 D	0.982 D	0.71 D	77 D
P139L	0.002 U	1.00 D	2.72 M	−8.79 D	−3.56 D	0.912 D	0.956 D	0.886 D	0.942 D	26.6 D	0.999 D	0.747 D	0.733 D	0.888 D	0.855 D	0.85 D	59 D
C151Y	0.001 U	1.00 D	2.745 M	−10.41 D	−3.34 D	0.925 D	1.005 D	0.900 D	0.923 D	25.2 D	0.996 D	0.916 D	0.939 D	0.988 D	0.939 D	0.9 D	82 D
T175M	0.002 U	0.940 D	1.98 M	−4.76 D	−2.48 D	0.774 D	0.798 D	0.812 D	0.891 D	33 D	0.999 D	0.685 D	0.242 N	0.833 D	0.719 D	0.7 D	20 D
G203S	0.001 U	0.997 D	3.865 H	−5.4 D	−7.34 D	0.737 D	0.915 D	0.966 D	0.966 D	27 D	0.998 D	0.826 D	0.898 D	0.909 D	0.909 D	0.89 D	77 D
G214R	0.001 U	1.00 D	4.2 H	−6.2 D	−6.13 D	0.965 D	0.997 D	0.989 D	0.949 D	26.7 D	0.999 D	0.934 D	0.924 D	0.98 D	0.904 D	0.9 D	94 D
R193W	0.071 U	1.00 D	1.755 M	−5.69 D	−2.45 D	0.805 D	0.068 D	0.733 D	0.806 D	27 D	0.998 D	0.240 D	0.566 D	0.909 D	0.834 D	0.63 D	55 D
F218L	0.001 D	0.990 D	2.915 M	−4.82 D	−3.25 D	0.766 D	0.898 D	0.861 D	0.807 D	24.7 D	0.998 D	0.904 D	0.769 D	0.531 D	0.899 D	0.84 D	76 D

AAS: amino acid substitutions. Mutation Taster: < 0.5. CADD: > 15. METALR: > 0.5. MCAP: > 0.025. PANTHER: probably damaging time > 450 my; possibly damaging, 450 my > time > 200 my; probably benign time < 200 my. VEST3: > 0.5. LRT: > 0.001. PROVEAN: > −2.667. FATHMM-MKK: < 0.5. PhD-SNP: > 0.5. SNP-GO: > 0.5. SNAP2: −100 (fully neutral); +100 (strong effect). DANN: > 0.5. Mutation Assessor: > 0.65 (−5.545 to 5.975 (higher score −> more damaging)). FATHMM: > 0.453.

**Table 3:** TM‐score and RMSD values of 7 selected damaging nsSNPs in *ELANE*.

SNP-ID	Residual change	TM-score	RMSD values
rs201163886	R34W	0.86482	1.98
rs28931611	C71Y	0.85993	2.05
rs201723157	R193W	0.95176	0.96
rs201139487	G203S	0.99524	0.49
rs137854451	G214R	0.96114	1.12
rs137854448	P139L	0.99994	0.04
rs200384291	F218L	0.87828	1.96

## Data Availability

All the data analyzed during the study are included with links in the paper.

## Supplementary Materials

Supplementary Materials Supplementary File 1: Table 1: twenty-one SIFT deleterious nsSNPs predictions through different in silico software. Table 2: total 301 ELANE missense SNPs SIFT prediction results. Supplementary File 2: Figure 1: overall significance of the prediction tools used in the study (the significance of the different prediction tools used in the study). Supplementary File 3: the effects of 50 highly risk pathogenic nsSNPs of ELANE gene on protein stability predicted by I-Mutant and MUpro (the effect of deleterious nsSNPs by two tools on protein stability and also the nsSNPs multiple allele frequency). Supplementary File 4: pathogenic nsSNPs analysis through HOPE Project. Supplementary File 5: Table 1: prediction of phosphorylation sites by NetPhos 3.1. Table 2: NetOGlyc 4.0 results for ELANE (wild type and final selected mutants). Supplementary File 6: Ramachandran plot and Chi1-chi2 plots of wild-type and mutant models.
