# Supplementary material for: In Silico Analysis Revealed Five Novel High-Risk Single-Nucleotide Polymorphisms (rs200384291, rs201163886, rs193141883, rs201139487, and rs201723157) in ELANE Gene Causing Autosomal Dominant Severe Congenital Neutropenia 1 and Cyclic Hematopoiesis
Source: ScientificWorldJournal. 2022 May 6;2022:3356835. doi: 10.1155/2022/3356835 (PMC9106522; doi:10.1155/2022/3356835)
Supplement: Supplementary Materials — Supplementary File 1: Table 1: twenty-one SIFT deleterious nsSNPs predictions through different in silico software. Table 2: total 301 ELANE missense SNPs SIFT prediction results. Supplementary File 2: Figure 1: overall significance of the prediction tools used in the study (the significance of the different prediction tools used in the study). Supplementary File 3: the effects of 50 highly risk pathogenic nsSNPs of ELANE gene on protein stability predicted by I-Mutant and MUpro (the effect of deleterious nsSNPs by two tools on protein stability and also the nsSNPs multiple allele frequency). Supplementary File 4: pathogenic nsSNPs analysis through HOPE Project. Supplementary File 5: Table 1: prediction of phosphorylation sites by NetPhos 3.1. Table 2: NetOGlyc 4.0 results for ELANE (wild type and final selected mutants). Supplementary File 6: Ramachandran plot and Chi1-chi2 plots of wild-type and mutant models. [file 3356835.f1.zip › 3356835.f1/S5.docx]

S5 Table 1: Prediction of Phosphorylation Sites by NetPhos 3.1

Table 1: Netphos-3.1b prediction results

#

# Sequence # x Context Score Kinase Answer

# -------------------------------------------------------------------

# Sequence 2 T ---MTLGRR 0.625 PKC YES

# Sequence 2 T ---MTLGRR 0.449 GSK3 .

# Sequence 2 T ---MTLGRR 0.410 cdc2 .

# Sequence 2 T ---MTLGRR 0.400 DNAPK .

# Sequence 2 T ---MTLGRR 0.397 CaM-II .

# Sequence 2 T ---MTLGRR 0.392 CKI .

# Sequence 2 T ---MTLGRR 0.371 PKA .

# Sequence 2 T ---MTLGRR 0.354 p38MAPK .

# Sequence 2 T ---MTLGRR 0.272 PKG .

# Sequence 2 T ---MTLGRR 0.269 cdk5 .

# Sequence 2 T ---MTLGRR 0.247 CKII .

# Sequence 2 T ---MTLGRR 0.229 ATM .

# Sequence 2 T ---MTLGRR 0.184 RSK .

# Sequence 2 T ---MTLGRR 0.173 unsp .

# Sequence 2 T ---MTLGRR 0.085 PKB .

#

# Sequence 24 T LLGGTALAS 0.454 CaM-II .

# Sequence 24 T LLGGTALAS 0.452 GSK3 .

# Sequence 24 T LLGGTALAS 0.427 cdc2 .

# Sequence 24 T LLGGTALAS 0.371 CKI .

# Sequence 24 T LLGGTALAS 0.349 CKII .

# Sequence 24 T LLGGTALAS 0.338 DNAPK .

# Sequence 24 T LLGGTALAS 0.296 p38MAPK .

# Sequence 24 T LLGGTALAS 0.276 PKG .

# Sequence 24 T LLGGTALAS 0.273 ATM .

# Sequence 24 T LLGGTALAS 0.193 RSK .

# Sequence 24 T LLGGTALAS 0.166 PKC .

# Sequence 24 T LLGGTALAS 0.144 cdk5 .

# Sequence 24 T LLGGTALAS 0.117 PKA .

# Sequence 24 T LLGGTALAS 0.082 PKB .

# Sequence 24 T LLGGTALAS 0.010 unsp .

#

# Sequence 28 S TALASEIVG 0.500 cdc2 .

# Sequence 28 S TALASEIVG 0.435 CaM-II .

# Sequence 28 S TALASEIVG 0.426 GSK3 .

# Sequence 28 S TALASEIVG 0.361 CKII .

# Sequence 28 S TALASEIVG 0.360 CKI .

# Sequence 28 S TALASEIVG 0.350 p38MAPK .

# Sequence 28 S TALASEIVG 0.339 DNAPK .

# Sequence 28 S TALASEIVG 0.335 PKG .

# Sequence 28 S TALASEIVG 0.306 PKC .

# Sequence 28 S TALASEIVG 0.289 ATM .

# Sequence 28 S TALASEIVG 0.259 RSK .

# Sequence 28 S TALASEIVG 0.242 cdk5 .

# Sequence 28 S TALASEIVG 0.129 PKA .

# Sequence 28 S TALASEIVG 0.105 unsp .

# Sequence 28 S TALASEIVG 0.084 PKB .

#

# Sequence 46 S PFMVSLQLR 0.506 PKA YES

# Sequence 46 S PFMVSLQLR 0.434 CaM-II .

# Sequence 46 S PFMVSLQLR 0.433 GSK3 .

# Sequence 46 S PFMVSLQLR 0.372 DNAPK .

# Sequence 46 S PFMVSLQLR 0.364 cdc2 .

# Sequence 46 S PFMVSLQLR 0.357 CKI .

# Sequence 46 S PFMVSLQLR 0.347 PKC .

# Sequence 46 S PFMVSLQLR 0.304 ATM .

# Sequence 46 S PFMVSLQLR 0.284 cdk5 .

# Sequence 46 S PFMVSLQLR 0.281 CKII .

# Sequence 46 S PFMVSLQLR 0.276 PKG .

# Sequence 46 S PFMVSLQLR 0.275 p38MAPK .

# Sequence 46 S PFMVSLQLR 0.254 RSK .

# Sequence 46 S PFMVSLQLR 0.084 PKB .

# Sequence 46 S PFMVSLQLR 0.002 unsp .

#

# Sequence 58 T FCGATLIAP 0.533 PKC YES

# Sequence 58 T FCGATLIAP 0.460 cdc2 .

# Sequence 58 T FCGATLIAP 0.448 CaM-II .

# Sequence 58 T FCGATLIAP 0.435 GSK3 .

# Sequence 58 T FCGATLIAP 0.356 CKI .

# Sequence 58 T FCGATLIAP 0.346 DNAPK .

# Sequence 58 T FCGATLIAP 0.343 PKA .

# Sequence 58 T FCGATLIAP 0.332 p38MAPK .

# Sequence 58 T FCGATLIAP 0.253 ATM .

# Sequence 58 T FCGATLIAP 0.252 CKII .

# Sequence 58 T FCGATLIAP 0.251 PKG .

# Sequence 58 T FCGATLIAP 0.233 RSK .

# Sequence 58 T FCGATLIAP 0.209 cdk5 .

# Sequence 58 T FCGATLIAP 0.086 PKB .

# Sequence 58 T FCGATLIAP 0.017 unsp .

#

# Sequence 67 S NFVMSAAHC 0.514 PKG YES

# Sequence 67 S NFVMSAAHC 0.451 cdc2 .

# Sequence 67 S NFVMSAAHC 0.444 GSK3 .

# Sequence 67 S NFVMSAAHC 0.438 CaM-II .

# Sequence 67 S NFVMSAAHC 0.395 DNAPK .

# Sequence 67 S NFVMSAAHC 0.364 CKI .

# Sequence 67 S NFVMSAAHC 0.357 p38MAPK .

# Sequence 67 S NFVMSAAHC 0.315 CKII .

# Sequence 67 S NFVMSAAHC 0.274 cdk5 .

# Sequence 67 S NFVMSAAHC 0.274 PKA .

# Sequence 67 S NFVMSAAHC 0.270 ATM .

# Sequence 67 S NFVMSAAHC 0.265 RSK .

# Sequence 67 S NFVMSAAHC 0.083 PKB .

# Sequence 67 S NFVMSAAHC 0.082 PKC .

# Sequence 67 S NFVMSAAHC 0.011 unsp .

#

# Sequence 90 S AHNLSRREP 0.989 unsp YES

# Sequence 90 S AHNLSRREP 0.482 CKII .

# Sequence 90 S AHNLSRREP 0.467 GSK3 .

# Sequence 90 S AHNLSRREP 0.425 PKA .

# Sequence 90 S AHNLSRREP 0.416 cdc2 .

# Sequence 90 S AHNLSRREP 0.415 CaM-II .

# Sequence 90 S AHNLSRREP 0.378 CKI .

# Sequence 90 S AHNLSRREP 0.356 DNAPK .

# Sequence 90 S AHNLSRREP 0.319 p38MAPK .

# Sequence 90 S AHNLSRREP 0.311 ATM .

# Sequence 90 S AHNLSRREP 0.311 PKG .

# Sequence 90 S AHNLSRREP 0.295 RSK .

# Sequence 90 S AHNLSRREP 0.253 PKC .

# Sequence 90 S AHNLSRREP 0.170 cdk5 .

# Sequence 90 S AHNLSRREP 0.081 PKB .

#

# Sequence 95 T RREPTRQVF 0.817 unsp YES

# Sequence 95 T RREPTRQVF 0.553 PKG YES

# Sequence 95 T RREPTRQVF 0.509 PKA YES

# Sequence 95 T RREPTRQVF 0.443 GSK3 .

# Sequence 95 T RREPTRQVF 0.437 CaM-II .

# Sequence 95 T RREPTRQVF 0.391 cdc2 .

# Sequence 95 T RREPTRQVF 0.359 CKI .

# Sequence 95 T RREPTRQVF 0.342 DNAPK .

# Sequence 95 T RREPTRQVF 0.294 RSK .

# Sequence 95 T RREPTRQVF 0.273 p38MAPK .

# Sequence 95 T RREPTRQVF 0.233 ATM .

# Sequence 95 T RREPTRQVF 0.229 CKII .

# Sequence 95 T RREPTRQVF 0.167 cdk5 .

# Sequence 95 T RREPTRQVF 0.130 PKB .

# Sequence 95 T RREPTRQVF 0.097 PKC .

#

# Sequence 109 Y FENGYDPVN 0.966 unsp YES

# Sequence 109 Y FENGYDPVN 0.520 INSR YES

# Sequence 109 Y FENGYDPVN 0.473 SRC .

# Sequence 109 Y FENGYDPVN 0.460 EGFR .

#

# Sequence 126 S QLNGSATIN 0.474 cdc2 .

# Sequence 126 S QLNGSATIN 0.444 GSK3 .

# Sequence 126 S QLNGSATIN 0.442 CaM-II .

# Sequence 126 S QLNGSATIN 0.433 DNAPK .

# Sequence 126 S QLNGSATIN 0.380 CKI .

# Sequence 126 S QLNGSATIN 0.363 PKG .

# Sequence 126 S QLNGSATIN 0.333 RSK .

# Sequence 126 S QLNGSATIN 0.321 ATM .

# Sequence 126 S QLNGSATIN 0.302 PKA .

# Sequence 126 S QLNGSATIN 0.287 p38MAPK .

# Sequence 126 S QLNGSATIN 0.274 CKII .

# Sequence 126 S QLNGSATIN 0.157 cdk5 .

# Sequence 126 S QLNGSATIN 0.084 PKC .

# Sequence 126 S QLNGSATIN 0.079 PKB .

# Sequence 126 S QLNGSATIN 0.009 unsp .

#

# Sequence 128 T NGSATINAN 0.445 CaM-II .

# Sequence 128 T NGSATINAN 0.416 PKC .

# Sequence 128 T NGSATINAN 0.409 GSK3 .

# Sequence 128 T NGSATINAN 0.366 cdc2 .

# Sequence 128 T NGSATINAN 0.362 CKI .

# Sequence 128 T NGSATINAN 0.349 DNAPK .

# Sequence 128 T NGSATINAN 0.319 p38MAPK .

# Sequence 128 T NGSATINAN 0.285 CKII .

# Sequence 128 T NGSATINAN 0.262 ATM .

# Sequence 128 T NGSATINAN 0.191 PKG .

# Sequence 128 T NGSATINAN 0.184 RSK .

# Sequence 128 T NGSATINAN 0.150 cdk5 .

# Sequence 128 T NGSATINAN 0.104 PKA .

# Sequence 128 T NGSATINAN 0.087 PKB .

# Sequence 128 T NGSATINAN 0.040 unsp .

#

# Sequence 167 S RGIASVLQE 0.613 PKC YES

# Sequence 167 S RGIASVLQE 0.484 PKA .

# Sequence 167 S RGIASVLQE 0.482 CaM-II .

# Sequence 167 S RGIASVLQE 0.471 cdc2 .

# Sequence 167 S RGIASVLQE 0.427 GSK3 .

# Sequence 167 S RGIASVLQE 0.418 CKII .

# Sequence 167 S RGIASVLQE 0.358 p38MAPK .

# Sequence 167 S RGIASVLQE 0.357 CKI .

# Sequence 167 S RGIASVLQE 0.352 DNAPK .

# Sequence 167 S RGIASVLQE 0.279 ATM .

# Sequence 167 S RGIASVLQE 0.271 RSK .

# Sequence 167 S RGIASVLQE 0.227 PKG .

# Sequence 167 S RGIASVLQE 0.212 cdk5 .

# Sequence 167 S RGIASVLQE 0.085 PKB .

# Sequence 167 S RGIASVLQE 0.008 unsp .

#

# Sequence 175 T ELNVTVVTS 0.460 cdc2 .

# Sequence 175 T ELNVTVVTS 0.455 CaM-II .

# Sequence 175 T ELNVTVVTS 0.442 GSK3 .

# Sequence 175 T ELNVTVVTS 0.364 CKI .

# Sequence 175 T ELNVTVVTS 0.347 p38MAPK .

# Sequence 175 T ELNVTVVTS 0.339 DNAPK .

# Sequence 175 T ELNVTVVTS 0.299 CKII .

# Sequence 175 T ELNVTVVTS 0.277 PKG .

# Sequence 175 T ELNVTVVTS 0.264 ATM .

# Sequence 175 T ELNVTVVTS 0.187 RSK .

# Sequence 175 T ELNVTVVTS 0.176 PKA .

# Sequence 175 T ELNVTVVTS 0.164 cdk5 .

# Sequence 175 T ELNVTVVTS 0.097 PKC .

# Sequence 175 T ELNVTVVTS 0.085 PKB .

# Sequence 175 T ELNVTVVTS 0.013 unsp .

#

# Sequence 178 T VTVVTSLCR 0.443 CaM-II .

# Sequence 178 T VTVVTSLCR 0.433 PKC .

# Sequence 178 T VTVVTSLCR 0.426 GSK3 .

# Sequence 178 T VTVVTSLCR 0.385 cdc2 .

# Sequence 178 T VTVVTSLCR 0.371 PKG .

# Sequence 178 T VTVVTSLCR 0.363 CKI .

# Sequence 178 T VTVVTSLCR 0.337 DNAPK .

# Sequence 178 T VTVVTSLCR 0.305 CKII .

# Sequence 178 T VTVVTSLCR 0.305 p38MAPK .

# Sequence 178 T VTVVTSLCR 0.224 ATM .

# Sequence 178 T VTVVTSLCR 0.204 RSK .

# Sequence 178 T VTVVTSLCR 0.181 cdk5 .

# Sequence 178 T VTVVTSLCR 0.117 PKA .

# Sequence 178 T VTVVTSLCR 0.078 PKB .

# Sequence 178 T VTVVTSLCR 0.050 unsp .

#

# Sequence 179 S TVVTSLCRR 0.468 GSK3 .

# Sequence 179 S TVVTSLCRR 0.439 cdc2 .

# Sequence 179 S TVVTSLCRR 0.420 PKC .

# Sequence 179 S TVVTSLCRR 0.417 CaM-II .

# Sequence 179 S TVVTSLCRR 0.377 DNAPK .

# Sequence 179 S TVVTSLCRR 0.363 CKI .

# Sequence 179 S TVVTSLCRR 0.338 p38MAPK .

# Sequence 179 S TVVTSLCRR 0.299 cdk5 .

# Sequence 179 S TVVTSLCRR 0.298 CKII .

# Sequence 179 S TVVTSLCRR 0.295 PKG .

# Sequence 179 S TVVTSLCRR 0.290 ATM .

# Sequence 179 S TVVTSLCRR 0.273 RSK .

# Sequence 179 S TVVTSLCRR 0.207 PKA .

# Sequence 179 S TVVTSLCRR 0.120 unsp .

# Sequence 179 S TVVTSLCRR 0.073 PKB .

#

# Sequence 184 S LCRRSNVCT 0.899 unsp YES

# Sequence 184 S LCRRSNVCT 0.742 PKA YES

# Sequence 184 S LCRRSNVCT 0.452 CaM-II .

# Sequence 184 S LCRRSNVCT 0.441 cdc2 .

# Sequence 184 S LCRRSNVCT 0.439 PKG .

# Sequence 184 S LCRRSNVCT 0.436 GSK3 .

# Sequence 184 S LCRRSNVCT 0.367 CKI .

# Sequence 184 S LCRRSNVCT 0.352 DNAPK .

# Sequence 184 S LCRRSNVCT 0.350 p38MAPK .

# Sequence 184 S LCRRSNVCT 0.290 RSK .

# Sequence 184 S LCRRSNVCT 0.273 ATM .

# Sequence 184 S LCRRSNVCT 0.233 CKII .

# Sequence 184 S LCRRSNVCT 0.198 cdk5 .

# Sequence 184 S LCRRSNVCT 0.087 PKB .

# Sequence 184 S LCRRSNVCT 0.085 PKC .

#

# Sequence 188 T SNVCTLVRG 0.464 GSK3 .

# Sequence 188 T SNVCTLVRG 0.417 CaM-II .

# Sequence 188 T SNVCTLVRG 0.409 cdc2 .

# Sequence 188 T SNVCTLVRG 0.376 cdk5 .

# Sequence 188 T SNVCTLVRG 0.368 CKI .

# Sequence 188 T SNVCTLVRG 0.352 DNAPK .

# Sequence 188 T SNVCTLVRG 0.318 p38MAPK .

# Sequence 188 T SNVCTLVRG 0.300 PKG .

# Sequence 188 T SNVCTLVRG 0.275 CKII .

# Sequence 188 T SNVCTLVRG 0.227 ATM .

# Sequence 188 T SNVCTLVRG 0.221 RSK .

# Sequence 188 T SNVCTLVRG 0.200 PKC .

# Sequence 188 T SNVCTLVRG 0.172 PKB .

# Sequence 188 T SNVCTLVRG 0.094 PKA .

# Sequence 188 T SNVCTLVRG 0.074 unsp .

#

# Sequence 202 S CFGDSGSPL 0.587 PKA YES

# Sequence 202 S CFGDSGSPL 0.466 CaM-II .

# Sequence 202 S CFGDSGSPL 0.461 GSK3 .

# Sequence 202 S CFGDSGSPL 0.429 cdc2 .

# Sequence 202 S CFGDSGSPL 0.360 CKI .

# Sequence 202 S CFGDSGSPL 0.345 DNAPK .

# Sequence 202 S CFGDSGSPL 0.311 PKG .

# Sequence 202 S CFGDSGSPL 0.311 CKII .

# Sequence 202 S CFGDSGSPL 0.274 p38MAPK .

# Sequence 202 S CFGDSGSPL 0.273 ATM .

# Sequence 202 S CFGDSGSPL 0.272 RSK .

# Sequence 202 S CFGDSGSPL 0.178 cdk5 .

# Sequence 202 S CFGDSGSPL 0.097 PKB .

# Sequence 202 S CFGDSGSPL 0.082 unsp .

# Sequence 202 S CFGDSGSPL 0.065 PKC .

#

# Sequence 204 S GDSGSPLVC 0.504 cdc2 YES

# Sequence 204 S GDSGSPLVC 0.494 p38MAPK .

# Sequence 204 S GDSGSPLVC 0.486 GSK3 .

# Sequence 204 S GDSGSPLVC 0.431 CaM-II .

# Sequence 204 S GDSGSPLVC 0.382 CKI .

# Sequence 204 S GDSGSPLVC 0.352 DNAPK .

# Sequence 204 S GDSGSPLVC 0.341 CKII .

# Sequence 204 S GDSGSPLVC 0.323 cdk5 .

# Sequence 204 S GDSGSPLVC 0.306 ATM .

# Sequence 204 S GDSGSPLVC 0.264 RSK .

# Sequence 204 S GDSGSPLVC 0.226 PKG .

# Sequence 204 S GDSGSPLVC 0.148 PKA .

# Sequence 204 S GDSGSPLVC 0.080 PKB .

# Sequence 204 S GDSGSPLVC 0.079 PKC .

# Sequence 204 S GDSGSPLVC 0.006 unsp .

#

# Sequence 217 S HGIASFVRG 0.624 PKC YES

# Sequence 217 S HGIASFVRG 0.451 CaM-II .

# Sequence 217 S HGIASFVRG 0.439 GSK3 .

# Sequence 217 S HGIASFVRG 0.433 cdc2 .

# Sequence 217 S HGIASFVRG 0.367 CKI .

# Sequence 217 S HGIASFVRG 0.362 DNAPK .

# Sequence 217 S HGIASFVRG 0.319 p38MAPK .

# Sequence 217 S HGIASFVRG 0.311 CKII .

# Sequence 217 S HGIASFVRG 0.278 ATM .

# Sequence 217 S HGIASFVRG 0.261 RSK .

# Sequence 217 S HGIASFVRG 0.258 PKG .

# Sequence 217 S HGIASFVRG 0.209 cdk5 .

# Sequence 217 S HGIASFVRG 0.162 PKA .

# Sequence 217 S HGIASFVRG 0.160 unsp .

# Sequence 217 S HGIASFVRG 0.084 PKB .

#

# Sequence 225 S GGCASGLYP 0.475 GSK3 .

# Sequence 225 S GGCASGLYP 0.471 cdc2 .

# Sequence 225 S GGCASGLYP 0.463 CaM-II .

# Sequence 225 S GGCASGLYP 0.380 CKII .

# Sequence 225 S GGCASGLYP 0.371 p38MAPK .

# Sequence 225 S GGCASGLYP 0.361 CKI .

# Sequence 225 S GGCASGLYP 0.345 DNAPK .

# Sequence 225 S GGCASGLYP 0.328 PKA .

# Sequence 225 S GGCASGLYP 0.295 ATM .

# Sequence 225 S GGCASGLYP 0.290 PKC .

# Sequence 225 S GGCASGLYP 0.275 RSK .

# Sequence 225 S GGCASGLYP 0.251 PKG .

# Sequence 225 S GGCASGLYP 0.243 cdk5 .

# Sequence 225 S GGCASGLYP 0.207 PKB .

# Sequence 225 S GGCASGLYP 0.021 unsp .

#

# Sequence 228 Y ASGLYPDAF 0.419 SRC .

# Sequence 228 Y ASGLYPDAF 0.415 INSR .

# Sequence 228 Y ASGLYPDAF 0.339 EGFR .

# Sequence 228 Y ASGLYPDAF 0.237 unsp .

#

# Sequence 244 S NWIDSIIQR 0.457 CaM-II .

# Sequence 244 S NWIDSIIQR 0.426 GSK3 .

# Sequence 244 S NWIDSIIQR 0.412 CKII .

# Sequence 244 S NWIDSIIQR 0.370 cdc2 .

# Sequence 244 S NWIDSIIQR 0.368 PKC .

# Sequence 244 S NWIDSIIQR 0.365 CKI .

# Sequence 244 S NWIDSIIQR 0.348 DNAPK .

# Sequence 244 S NWIDSIIQR 0.302 ATM .

# Sequence 244 S NWIDSIIQR 0.297 PKG .

# Sequence 244 S NWIDSIIQR 0.295 p38MAPK .

# Sequence 244 S NWIDSIIQR 0.236 RSK .

# Sequence 244 S NWIDSIIQR 0.232 PKA .

# Sequence 244 S NWIDSIIQR 0.161 cdk5 .

# Sequence 244 S NWIDSIIQR 0.080 PKB .

# Sequence 244 S NWIDSIIQR 0.008 unsp .

#

# Sequence 249 S IIQRSEDNP 0.824 unsp YES

# Sequence 249 S IIQRSEDNP 0.497 CKII .

# Sequence 249 S IIQRSEDNP 0.467 CaM-II .

# Sequence 249 S IIQRSEDNP 0.457 cdc2 .

# Sequence 249 S IIQRSEDNP 0.447 GSK3 .

# Sequence 249 S IIQRSEDNP 0.376 CKI .

# Sequence 249 S IIQRSEDNP 0.356 RSK .

# Sequence 249 S IIQRSEDNP 0.350 DNAPK .

# Sequence 249 S IIQRSEDNP 0.314 p38MAPK .

# Sequence 249 S IIQRSEDNP 0.312 ATM .

# Sequence 249 S IIQRSEDNP 0.305 PKG .

# Sequence 249 S IIQRSEDNP 0.282 PKA .

# Sequence 249 S IIQRSEDNP 0.201 cdk5 .

# Sequence 249 S IIQRSEDNP 0.093 PKC .

# Sequence 249 S IIQRSEDNP 0.083 PKB .

#

# Sequence 264 S PDPASRTH- 0.469 GSK3 .

# Sequence 264 S PDPASRTH- 0.443 CaM-II .

# Sequence 264 S PDPASRTH- 0.420 cdc2 .

# Sequence 264 S PDPASRTH- 0.366 CKI .

# Sequence 264 S PDPASRTH- 0.362 CKII .

# Sequence 264 S PDPASRTH- 0.360 DNAPK .

# Sequence 264 S PDPASRTH- 0.353 ATM .

# Sequence 264 S PDPASRTH- 0.328 p38MAPK .

# Sequence 264 S PDPASRTH- 0.296 RSK .

# Sequence 264 S PDPASRTH- 0.274 PKA .

# Sequence 264 S PDPASRTH- 0.258 PKG .

# Sequence 264 S PDPASRTH- 0.218 cdk5 .

# Sequence 264 S PDPASRTH- 0.216 unsp .

# Sequence 264 S PDPASRTH- 0.201 PKC .

# Sequence 264 S PDPASRTH- 0.094 PKB .

#

# Sequence 266 T PASRTH--- 0.450 GSK3 .

# Sequence 266 T PASRTH--- 0.445 cdc2 .

# Sequence 266 T PASRTH--- 0.420 CaM-II .

# Sequence 266 T PASRTH--- 0.412 PKC .

# Sequence 266 T PASRTH--- 0.381 CKII .

# Sequence 266 T PASRTH--- 0.365 CKI .

# Sequence 266 T PASRTH--- 0.347 DNAPK .

# Sequence 266 T PASRTH--- 0.328 PKG .

# Sequence 266 T PASRTH--- 0.319 p38MAPK .

# Sequence 266 T PASRTH--- 0.270 ATM .

# Sequence 266 T PASRTH--- 0.261 RSK .

# Sequence 266 T PASRTH--- 0.198 cdk5 .

# Sequence 266 T PASRTH--- 0.113 PKA .

# Sequence 266 T PASRTH--- 0.076 PKB .

# Sequence 266 T PASRTH--- 0.052 unsp .

#

MTLGRRLACLFLACVLPALLLGGTALASEIVGGRRARPHAWPFMVSLQLR # 50

GGHFCGATLIAPNFVMSAAHCVANVNVRAVRVVLGAHNLSRREPTRQVFA # 100

VQRIFENGYDPVNLLNDIVILQLNGSATINANVQVAQLPAQGRRLGNGVQ # 150

CLAMGWGLLGRNRGIASVLQELNVTVVTSLCRRSNVCTLVRGRQAGVCFG # 200

DSGSPLVCNGLIHGIASFVRGGCASGLYPDAFAPVAQFVNWIDSIIQRSE # 250

DNPCPHPRDPDPASRTH # 300

%1 .T...........................................S.... # 50

%1 .......T........S......................S....T..... # 100

%1 ........Y......................................... # 150

%1 ................S................S................ # 200

%1 .S.S............S...............................S. # 250

%1 .................


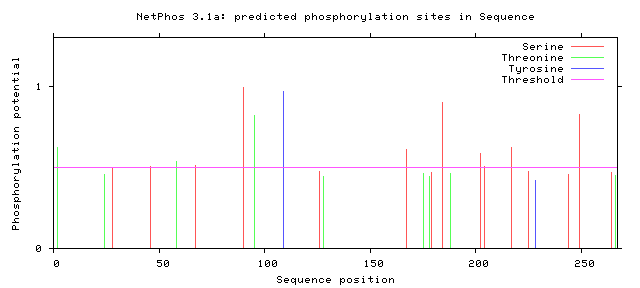


S5 Table 2: NetOGlyc 4.0 Results for ELANE (Wild type and final selected Mutants)

Table 2. Glycosylation of the protein

##gff-version 2

##source-version NetOGlyc 4.0.0.13

##date 21-7-11

##Type Protein

#seqname source feature start end score strand frame comment

SEQUENCE netOGlyc-4.0.0.13 CARBOHYD 2 2 0.0253987 . .

SEQUENCE netOGlyc-4.0.0.13 CARBOHYD 24 24 0.0685934 . .

SEQUENCE netOGlyc-4.0.0.13 CARBOHYD 28 28 0.244496 . .

SEQUENCE netOGlyc-4.0.0.13 CARBOHYD 46 46 0.0422618 . .

SEQUENCE netOGlyc-4.0.0.13 CARBOHYD 58 58 0.0237173 . .

SEQUENCE netOGlyc-4.0.0.13 CARBOHYD 67 67 0.0172228 . .

SEQUENCE netOGlyc-4.0.0.13 CARBOHYD 90 90 0.0826022 . .

SEQUENCE netOGlyc-4.0.0.13 CARBOHYD 95 95 0.131957 . .

SEQUENCE netOGlyc-4.0.0.13 CARBOHYD 126 126 0.0518992 . .

SEQUENCE netOGlyc-4.0.0.13 CARBOHYD 128 128 0.0522798 . .

SEQUENCE netOGlyc-4.0.0.13 CARBOHYD 167 167 0.12794 . .

SEQUENCE netOGlyc-4.0.0.13 CARBOHYD 175 175 0.0989285 . .

SEQUENCE netOGlyc-4.0.0.13 CARBOHYD 178 178 0.110544 . .

SEQUENCE netOGlyc-4.0.0.13 CARBOHYD 179 179 0.119155 . .

SEQUENCE netOGlyc-4.0.0.13 CARBOHYD 184 184 0.134773 . .

SEQUENCE netOGlyc-4.0.0.13 CARBOHYD 188 188 0.419767 . .

SEQUENCE netOGlyc-4.0.0.13 CARBOHYD 202 202 0.0427849 . .

SEQUENCE netOGlyc-4.0.0.13 CARBOHYD 204 204 0.0108217 . .

SEQUENCE netOGlyc-4.0.0.13 CARBOHYD 217 217 0.0259658 . .

SEQUENCE netOGlyc-4.0.0.13 CARBOHYD 225 225 0.117533 . .

SEQUENCE netOGlyc-4.0.0.13 CARBOHYD 244 244 0.0969857 . .

SEQUENCE netOGlyc-4.0.0.13 CARBOHYD 249 249 0.301325 . .

SEQUENCE netOGlyc-4.0.0.13 CARBOHYD 264 264 0.194582 . .

SEQUENCE netOGlyc-4.0.0.13 CARBOHYD 266 266 0.121188 . .

**Tabel3. Glycosylation after inserting the mutations in** protein

##gff-version 2

##source-version NetOGlyc 4.0.0.13

##date 21-7-11

##Type Protein

#seqname source feature start end score strand frame comment

SEQUENCE netOGlyc-4.0.0.13 CARBOHYD 2 2 0.0279924 . .

SEQUENCE netOGlyc-4.0.0.13 CARBOHYD 24 24 0.0684885 . .

SEQUENCE netOGlyc-4.0.0.13 CARBOHYD 28 28 0.232673 . .

SEQUENCE netOGlyc-4.0.0.13 CARBOHYD 46 46 0.0234982 . .

SEQUENCE netOGlyc-4.0.0.13 CARBOHYD 58 58 0.0118642 . .

SEQUENCE netOGlyc-4.0.0.13 CARBOHYD 67 67 0.0167754 . .

SEQUENCE netOGlyc-4.0.0.13 CARBOHYD 90 90 0.0674089 . .

SEQUENCE netOGlyc-4.0.0.13 CARBOHYD 95 95 0.109429 . .

SEQUENCE netOGlyc-4.0.0.13 CARBOHYD 126 126 0.0437499 . .

SEQUENCE netOGlyc-4.0.0.13 CARBOHYD 128 128 0.0438565 . .

SEQUENCE netOGlyc-4.0.0.13 CARBOHYD 167 167 0.118369 . .

SEQUENCE netOGlyc-4.0.0.13 CARBOHYD 175 175 0.0789128 . .

SEQUENCE netOGlyc-4.0.0.13 CARBOHYD 178 178 0.106684 . .

SEQUENCE netOGlyc-4.0.0.13 CARBOHYD 179 179 0.120236 . .

SEQUENCE netOGlyc-4.0.0.13 CARBOHYD 184 184 0.073349 . .

SEQUENCE netOGlyc-4.0.0.13 CARBOHYD 188 188 0.295532 . .

SEQUENCE netOGlyc-4.0.0.13 CARBOHYD 202 202 0.0198122 . .

SEQUENCE netOGlyc-4.0.0.13 CARBOHYD 203 203 0.0195638 . .

SEQUENCE netOGlyc-4.0.0.13 CARBOHYD 204 204 0.0117891 . .

SEQUENCE netOGlyc-4.0.0.13 CARBOHYD 217 217 0.0424571 . .

SEQUENCE netOGlyc-4.0.0.13 CARBOHYD 225 225 0.108665 . .

SEQUENCE netOGlyc-4.0.0.13 CARBOHYD 244 244 0.0787338 . .

SEQUENCE netOGlyc-4.0.0.13 CARBOHYD 249 249 0.259795 . .

SEQUENCE netOGlyc-4.0.0.13 CARBOHYD 264 264 0.20841 . .

SEQUENCE netOGlyc-4.0.0.13 CARBOHYD 266 266 0.123157 . .
