# Supplementary material for: In Silico Analysis Revealed Five Novel High-Risk Single-Nucleotide Polymorphisms (rs200384291, rs201163886, rs193141883, rs201139487, and rs201723157) in ELANE Gene Causing Autosomal Dominant Severe Congenital Neutropenia 1 and Cyclic Hematopoiesis
Source: ScientificWorldJournal. 2022 May 6;2022:3356835. doi: 10.1155/2022/3356835 (PMC9106522; doi:10.1155/2022/3356835)
Supplement: Supplementary Materials — Supplementary File 1: Table 1: twenty-one SIFT deleterious nsSNPs predictions through different in silico software. Table 2: total 301 ELANE missense SNPs SIFT prediction results. Supplementary File 2: Figure 1: overall significance of the prediction tools used in the study (the significance of the different prediction tools used in the study). Supplementary File 3: the effects of 50 highly risk pathogenic nsSNPs of ELANE gene on protein stability predicted by I-Mutant and MUpro (the effect of deleterious nsSNPs by two tools on protein stability and also the nsSNPs multiple allele frequency). Supplementary File 4: pathogenic nsSNPs analysis through HOPE Project. Supplementary File 5: Table 1: prediction of phosphorylation sites by NetPhos 3.1. Table 2: NetOGlyc 4.0 results for ELANE (wild type and final selected mutants). Supplementary File 6: Ramachandran plot and Chi1-chi2 plots of wild-type and mutant models. [file 3356835.f1.zip › 3356835.f1/S6 Ramachandran.docx]

**S6** [**Ramachandran plot**](https://www.google.com/search?sxsrf=ALeKk00miroJe-uui-FzQj4vwxmmQfw9Ig:1628058535123&q=Ramachandran+plot&spell=1&sa=X&ved=2ahUKEwjf46LV3pbyAhVJlYsKHdIqAK0QBSgAegQIARAx) **and Chi plot of Wild and mutant types**

**Wild type**

**
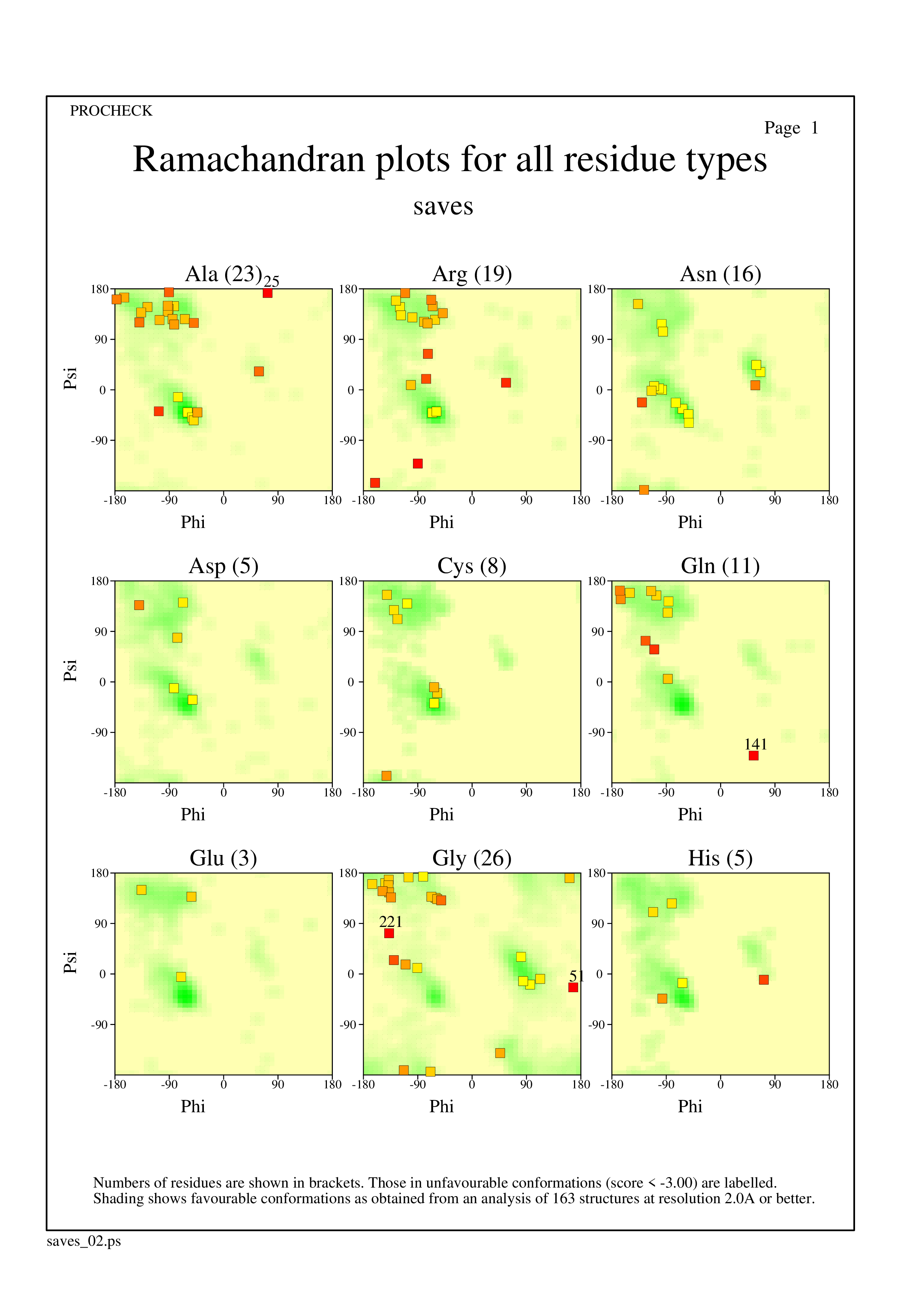
**

**
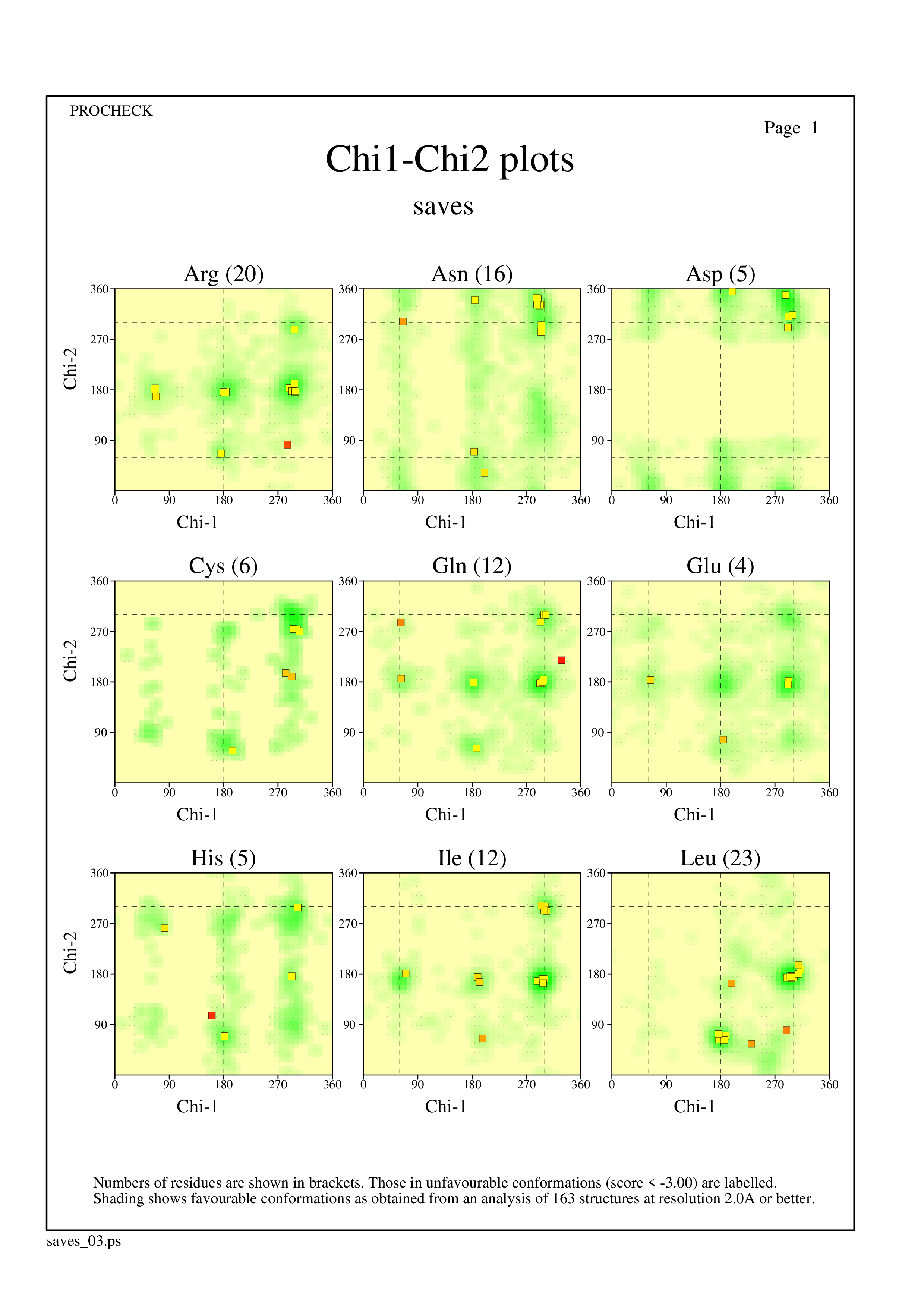
**

**F218l**

**
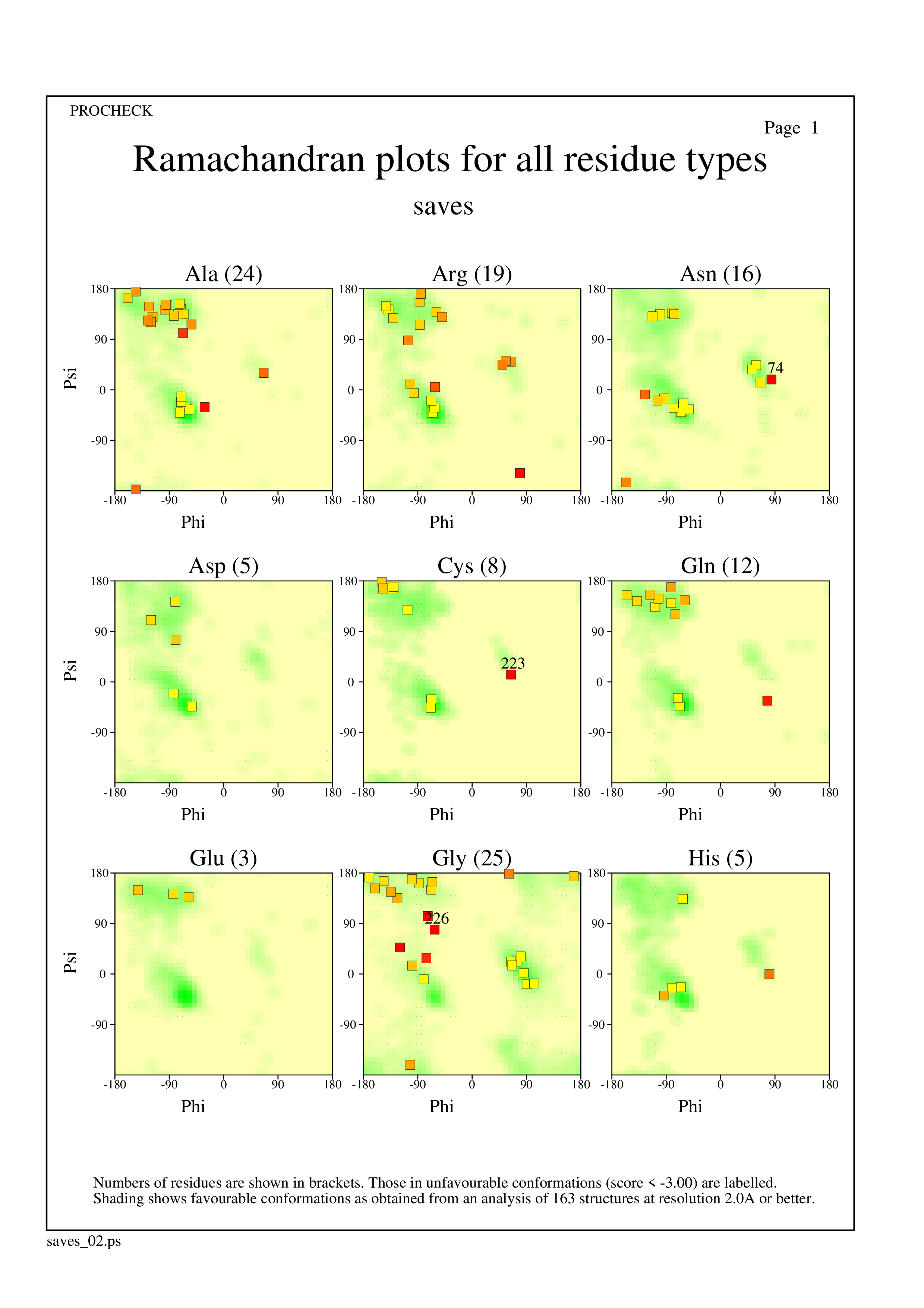
**

**
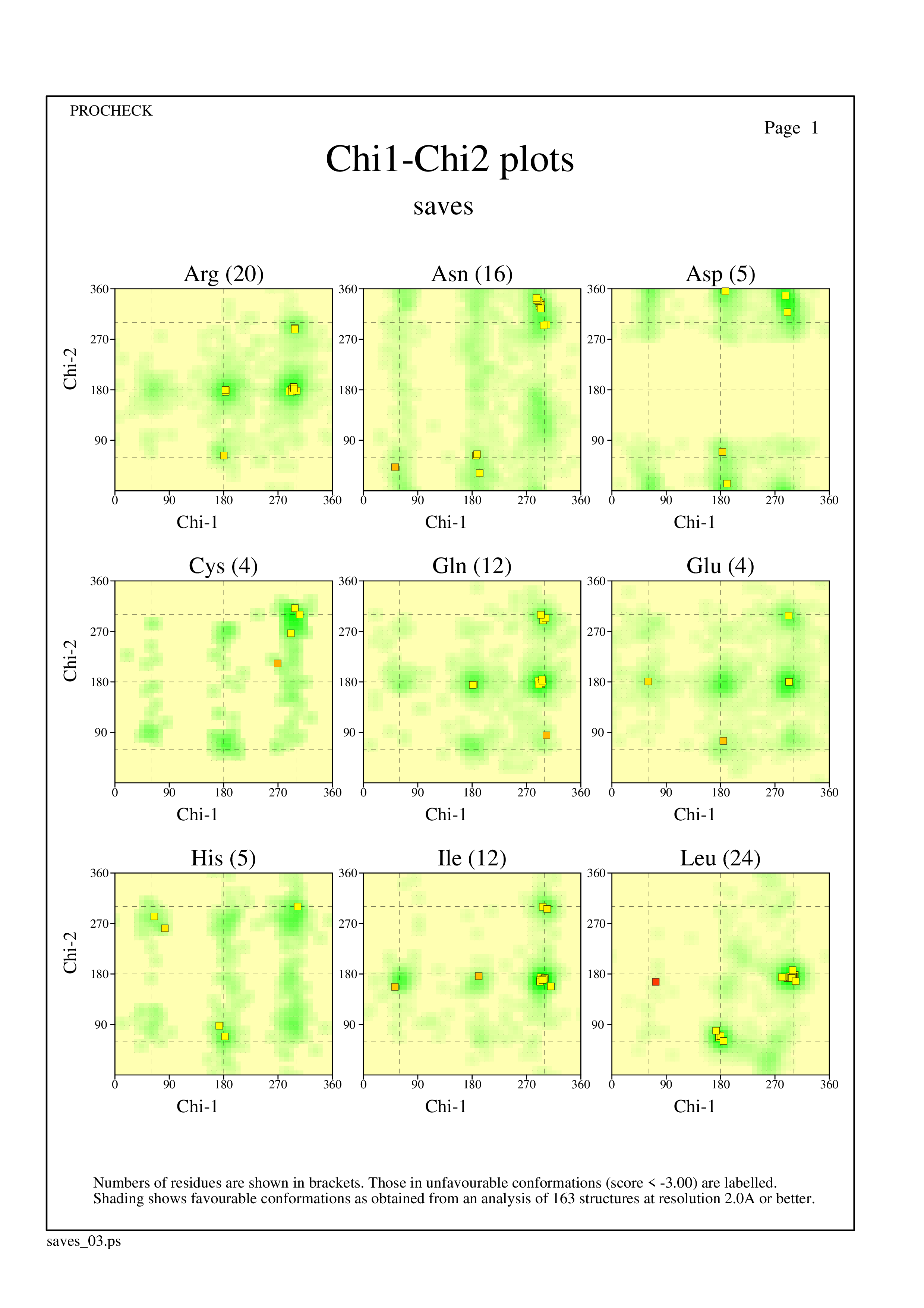
**

**C71Y**

**
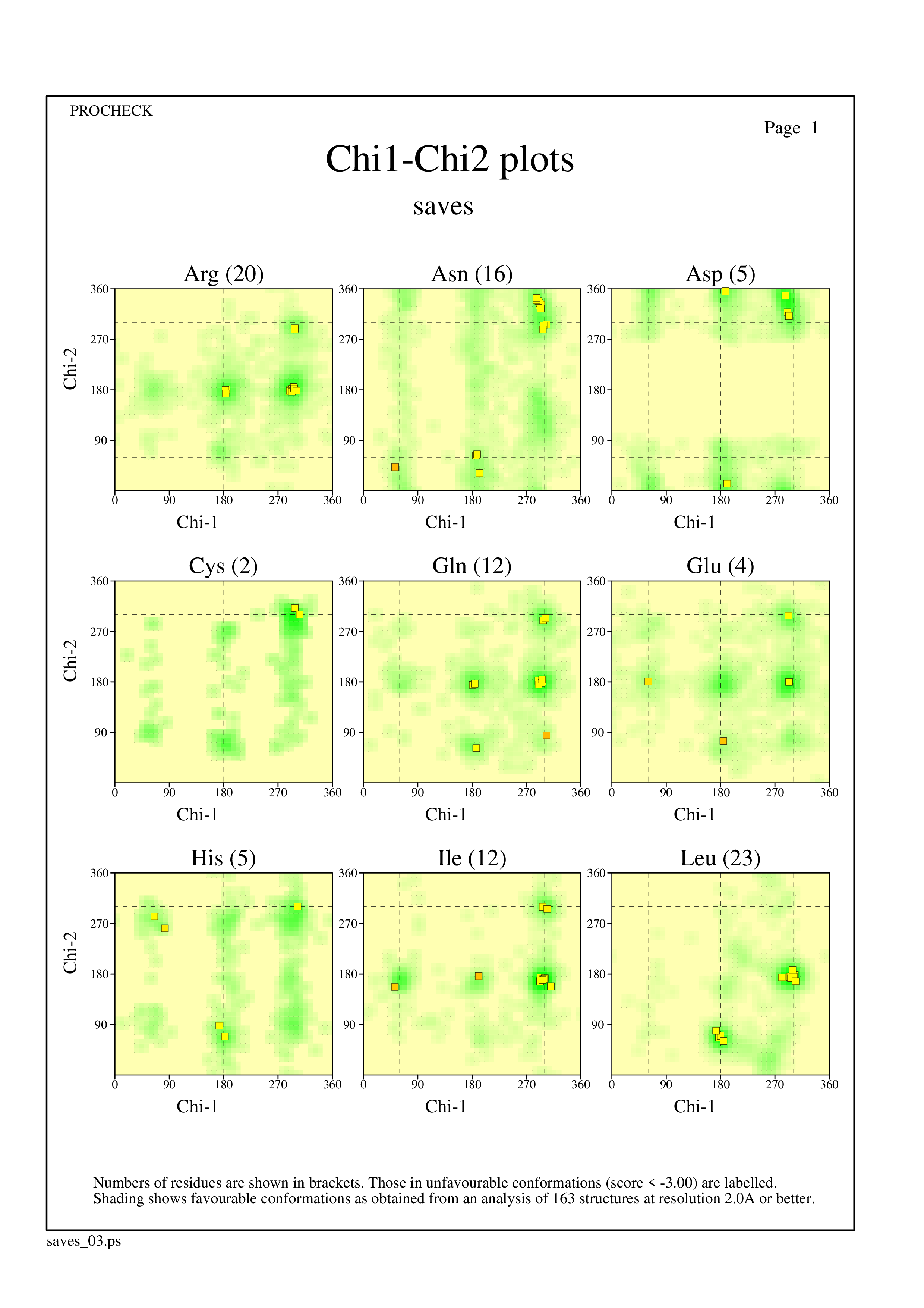
**

**
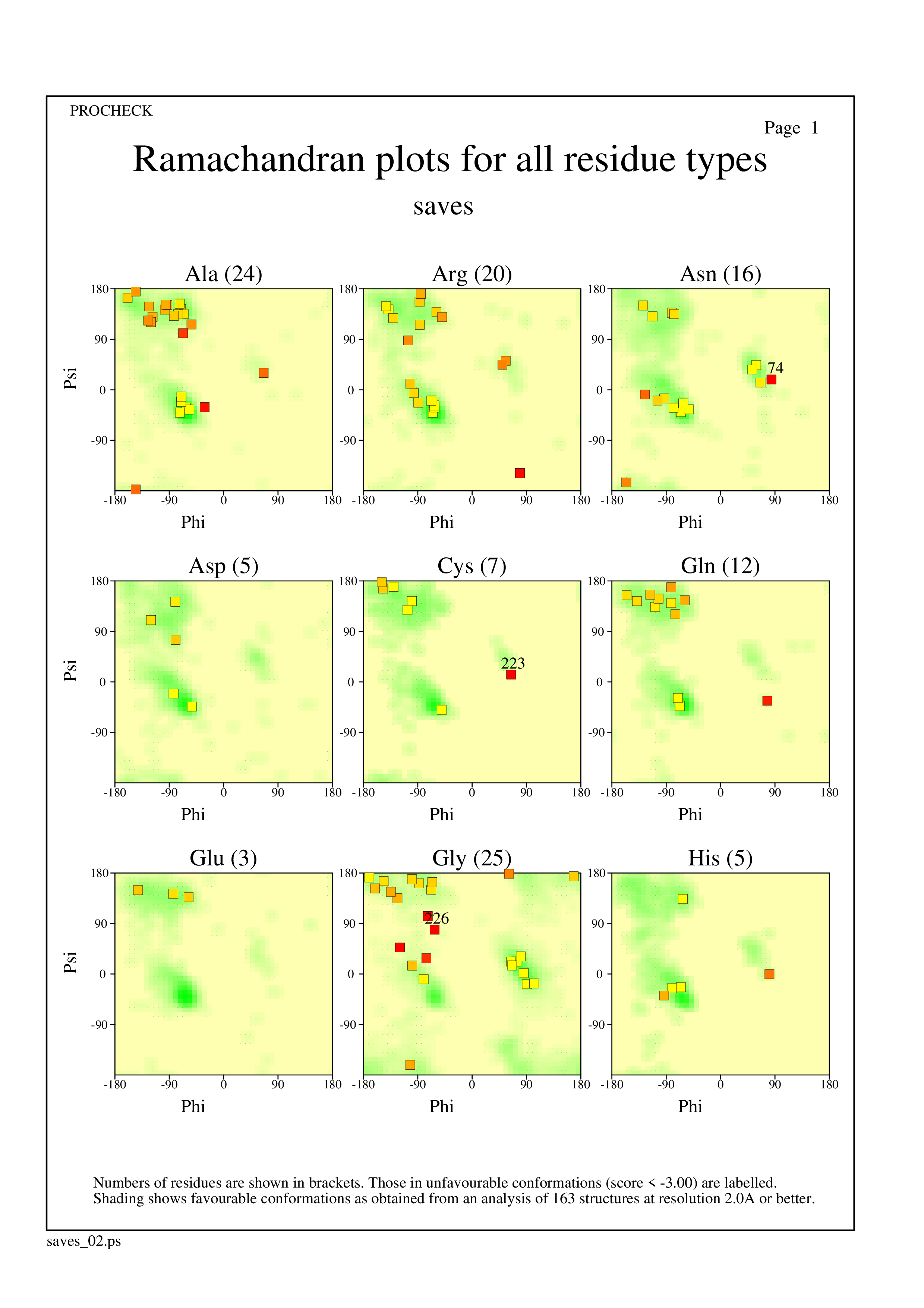
**

**G214R**

**
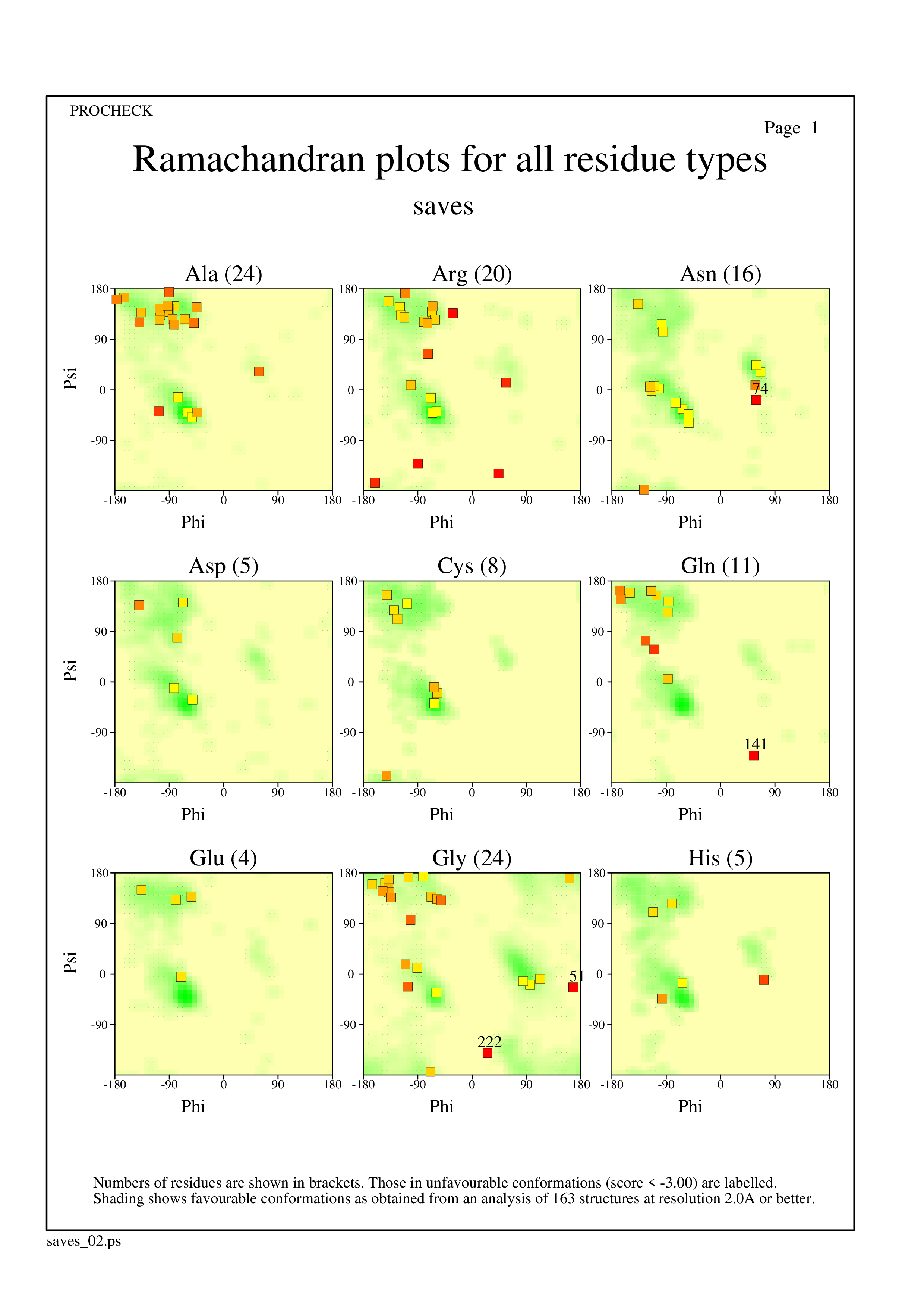
**

**
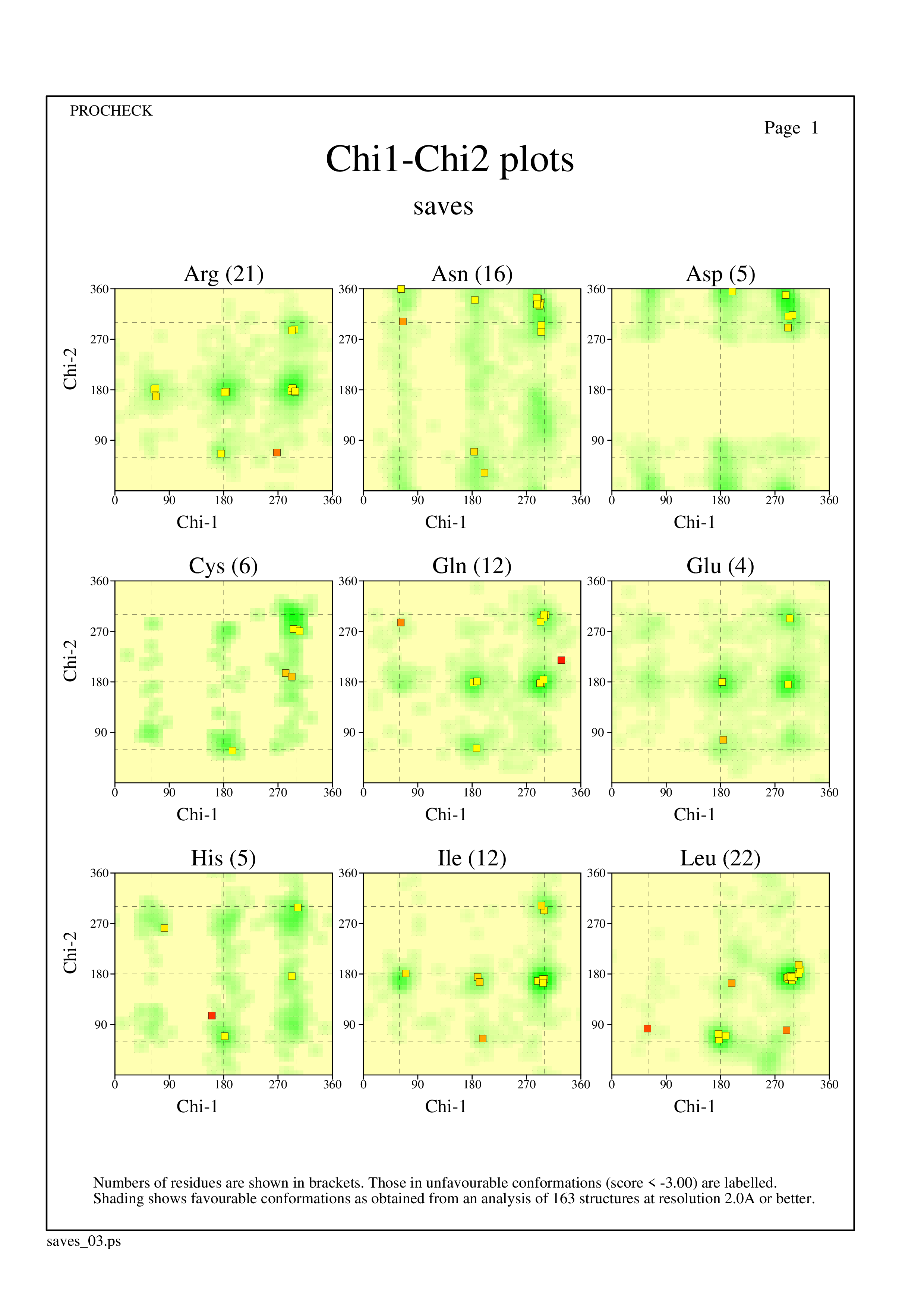
**

**R34W**

**
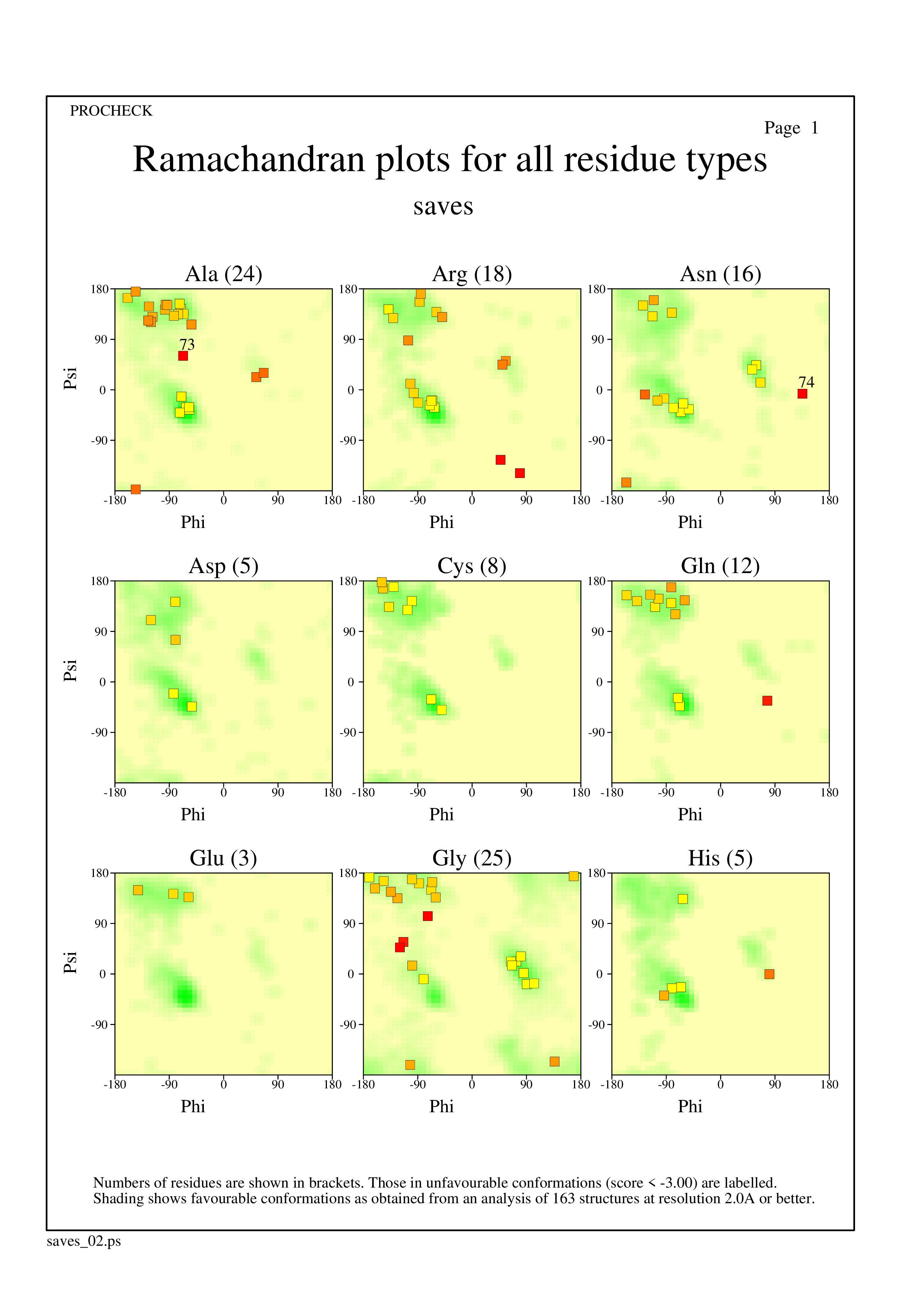
**

**
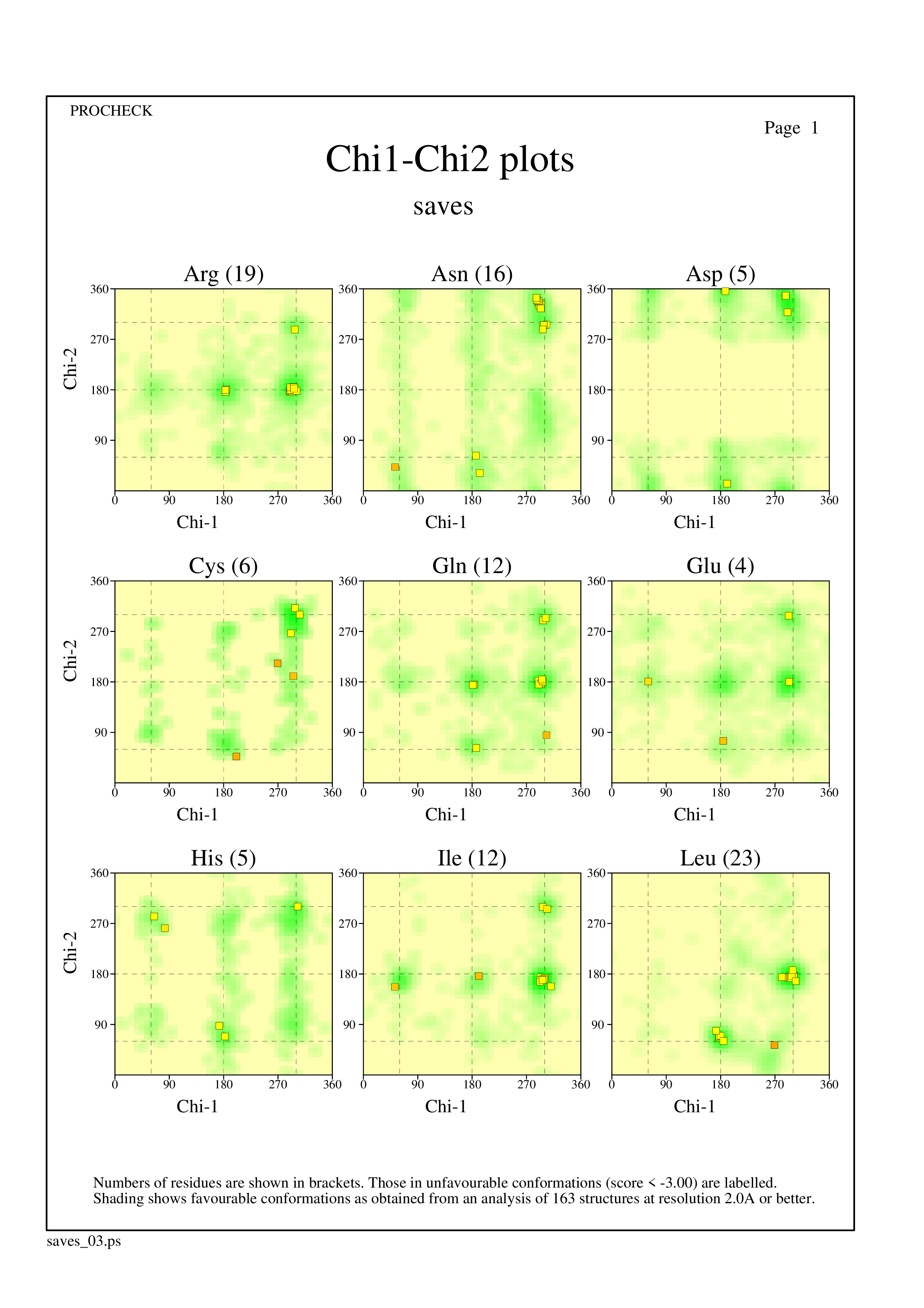
**
