# Supplementary material for: In Silico Analysis Revealed Five Novel High-Risk Single-Nucleotide Polymorphisms (rs200384291, rs201163886, rs193141883, rs201139487, and rs201723157) in ELANE Gene Causing Autosomal Dominant Severe Congenital Neutropenia 1 and Cyclic Hematopoiesis
Source: ScientificWorldJournal. 2022 May 6;2022:3356835. doi: 10.1155/2022/3356835 (PMC9106522; doi:10.1155/2022/3356835)
Supplement: Supplementary Materials — Supplementary File 1: Table 1: twenty-one SIFT deleterious nsSNPs predictions through different in silico software. Table 2: total 301 ELANE missense SNPs SIFT prediction results. Supplementary File 2: Figure 1: overall significance of the prediction tools used in the study (the significance of the different prediction tools used in the study). Supplementary File 3: the effects of 50 highly risk pathogenic nsSNPs of ELANE gene on protein stability predicted by I-Mutant and MUpro (the effect of deleterious nsSNPs by two tools on protein stability and also the nsSNPs multiple allele frequency). Supplementary File 4: pathogenic nsSNPs analysis through HOPE Project. Supplementary File 5: Table 1: prediction of phosphorylation sites by NetPhos 3.1. Table 2: NetOGlyc 4.0 results for ELANE (wild type and final selected mutants). Supplementary File 6: Ramachandran plot and Chi1-chi2 plots of wild-type and mutant models. [file 3356835.f1.zip › 3356835.f1/Supplementary file 3.docx]

| Supplementary file 3: The effects of high-risk nsSNPs of ELANE gene on protein stability predicted by I-Mutant 3.0, and MUpro tools. | | | |
| --- | --- | --- | --- |
| **high-risk nsSNP** | **I-Mutation 3.0** | **Mupro** |  |
| R34W | D -0.30 | -0.37609409 |  |
| C71R | D -0.43 | -0.37718502 |  |
| V101M | D -0.88 | -1.0951531 |  |
| V101L | I -0.60 | -0.92071308 |  |
| P139L | D -0.40 | 0.41684183 |  |
| R143C | D -0.59 | D-0.75874933 | |
| C151Y | D -0.07 | D-0.2124111 |  |
| A166T | D -0.72 | D-1.0287181 |  |
| A166V | I 0.13 | -0.40338027 |  |
| T175M | D -0.38 | -0.53590021 |  |
| R182H | D -1.49 | -1.0371122 |  |
| V190M | D -1.01 | -0.81657212 |  |
| R193W | D -0.47 | -0.61414549 |  |
| G203S | D -1.16 | -0.52045334 |  |
| L206F | D -1.26 | -0.32883401 |  |
| N209K | D -0.48 | -1.3473093 |  |
| G210R | D -0.74 | -0.82271872 |  |
| G214R | D -0.6 | -0.548992 |  |
| F218L | D -0.99 | -1.0285226 |  |
| P262S | D -1.25 |  |  |
| P262L | D -0.30 |  |  |
