# Supplementary material for: In Silico Analysis Revealed Five Novel High-Risk Single-Nucleotide Polymorphisms (rs200384291, rs201163886, rs193141883, rs201139487, and rs201723157) in ELANE Gene Causing Autosomal Dominant Severe Congenital Neutropenia 1 and Cyclic Hematopoiesis
Source: ScientificWorldJournal. 2022 May 6;2022:3356835. doi: 10.1155/2022/3356835 (PMC9106522; doi:10.1155/2022/3356835)
Supplement: Supplementary Materials — Supplementary File 1: Table 1: twenty-one SIFT deleterious nsSNPs predictions through different in silico software. Table 2: total 301 ELANE missense SNPs SIFT prediction results. Supplementary File 2: Figure 1: overall significance of the prediction tools used in the study (the significance of the different prediction tools used in the study). Supplementary File 3: the effects of 50 highly risk pathogenic nsSNPs of ELANE gene on protein stability predicted by I-Mutant and MUpro (the effect of deleterious nsSNPs by two tools on protein stability and also the nsSNPs multiple allele frequency). Supplementary File 4: pathogenic nsSNPs analysis through HOPE Project. Supplementary File 5: Table 1: prediction of phosphorylation sites by NetPhos 3.1. Table 2: NetOGlyc 4.0 results for ELANE (wild type and final selected mutants). Supplementary File 6: Ramachandran plot and Chi1-chi2 plots of wild-type and mutant models. [file 3356835.f1.zip › 3356835.f1/S4 projct hop.doc]

**Supplementary File 8.** Predicted effects of mutations from 15 high-risk pathogenic nsSNPs ELANE gene on size, charge, hydrophobicity, spatial structure and function of amino acids.

| Mutation | ConSurf  Score | Amino acid change | charge | Hydrophobicity | Overall Impact |
| --- | --- | --- | --- | --- | --- |
| R34W | 8 | W<M | Pos→Neu | ↑ | The mutant residue is bigger than the wild-type residue. The wild-type residue charge was POSITIVE, the mutant residue charge is NEUTRAL. The mutant residue is more hydrophobic than the wild-type residue. The wild-type and mutant amino acids differ in size. The mutant residue is bigger, this might lead to bumps. The hydrophobicity of the wild-type and mutant residue differs. |
| C71R | 9 | W<M | Neu →Pos - |  | The mutant residue is bigger than the wild-type residue. The wild-type residue charge was NEUTRAL, the mutant residue charge is POSITIVE. The wild-type residue is more hydrophobic than the mutant residue. The wild-type and mutant amino acids differ in size. The mutant residue is bigger, this might lead to bumps. The hydrophobicity of the wild-type and mutant residue differs. Hydrophobic interactions, either in the core of the protein or on the surface, will be lost. |
| V101M | 9 | W<M | - |  | The mutant residue is bigger than the wild-type residue. The mutated residue is located in a domain that is important for the main activity of the protein. Mutation of the residue might disturb this function. The wild-type and mutant amino acids differ in size. The mutant residue is bigger, this might lead to bumps. |
| P139L | 8 | W<M |  |  | The mutant residue is bigger than the wild-type residue. The mutated residue is located in a domain that is important for the main activity of the protein. Mutation of the residue might disturb this function. The wild-type and mutant amino acids differ in size. The mutant residue is bigger, this might lead to bumps. Prolines are known to have a very rigid structure, sometimes forcing the the backbone in a specific conformation. possibly, your mutation changes a proline with such a function into another residue, thereby disturbing the local structure |
| R143C | 9 | W>M | Pos→Neu - | ↑ | The mutant residue is smaller than the wild-type residue. The wild-type residue charge was POSITIVE, the mutant residue charge is NEUTRAL. The mutant residue is more hydrophobic than the wild-type residue. The mutation is located within a domain, annotated in UniProt as:Peptidase S1 |
| C151Y | 8 | W<M | - | ↑ | The mutant residue is bigger than the wild-type residue. The wild-type residue is more hydrophobic than the mutant residue. The mutation is located within a domain, annotated in UniProt as: Peptidase S1. The mutation introduces an amino acid with different properties, which can disturb this domain and abolish its function. The wild-type and mutant amino acids differ in size. The mutant residue is bigger, this might lead to bumps. The hydrophobicity of the wild-type and mutant residue differs. Hydrophobic interactions, either in the core of the protein or on the surface, will be lost. |
| A166T | 8 | W<M | Neu→Neg | ↑ | The mutant residue is bigger than the wild-type residue. The wild-type residue charge was NEUTRAL, the mutant residue charge is NEGATIVE. The wild-type residue is more hydrophobic than the mutant residue. There is a difference in charge between the wild-type and mutant amino acid. The mutation introduces a charge, this can cause repulsion of ligands or other residues with the same charge. The wild-type and mutant amino acids differ in size. The mutant residue is bigger, this might lead to bumps. The hydrophobicity of the wild-type and mutant residue differs. Hydrophobic interactions, either in the core of the protein or on the surface, will be lost. |
| A166V |  | W<M |  |  | You are interested in the mutation of a Alanine into a Valine at position 166. The mutation is located within a domain, annotated in UniProt as: Peptidase S1 The mutation introduces an amino acid with different properties, which can disturb this domain and abolish its function. The mutated residue is located in a domain that is important for the main activity of the protein. Mutation of the residue might disturb this function. The wild-type and mutant amino acids differ in size. The mutant residue is bigger, this might lead to bumps. |
| T175M | 9 | W<M |  | ↑ | You are interested in the mutation of a Threonine into a Methionine at position 175. The mutant residue is bigger than the wild-type residue. The mutant residue is more hydrophobic than the wild-type residue. The mutant residue is bigger than the wild-type residue. The mutant residue is more hydrophobic than the wild-type residue. The mutation is located within a domain, annotated in UniProt as: Peptidase S1. The mutation introduces an amino acid with different properties, which can disturb this domain and abolish its function. The wild-type and mutant amino acids differ in size. The mutant residue is bigger, this might lead to bumps. The hydrophobicity of the wild-type and mutant residue differs. |
| R182H | 9 | W>M | Pos→Neu | ↑ | The mutant residue is smaller than the wild-type residue. The wild-type residue charge was POSITIVE, the mutant residue charge is NEUTRAL. There is a difference in charge between the wild-type and mutant amino acid. The charge of the wild-type residue will be lost, this can cause loss of interactions with other molecules or residues. The wild-type and mutant amino acids differ in size. The mutant residue is smaller, this might lead to loss of interactions. |
| V190M | 9 | W<M |  |  | The mutant residue is bigger than the wild-type residue. The mutation is located within a domain, annotated in UniProt as: Peptidase S1. The wild-type and mutant amino acids differ in size. The mutant residue is bigger, this might lead to bumps. |
| R193W | 9 |  |  | ↑ | The mutant residue is more hydrophobic than the wild-type residue. The hydrophobicity of the wild-type and mutant residue differs. The mutation introduces a more hydrophobic residue at this position. This can result in loss of hydrogen bonds and/or disturb correct folding. |
| G203S | 9 | W<M | - |  | The mutant residue is bigger than the wild-type residue. The mutant residue is bigger, this might lead to bumps. The wild-type and mutant amino acids differ in size. The mutant residue is bigger, this might lead to bumps. |
| L206F | 9 | W<M |  |  | The mutant residue is bigger than the wild-type residue. The wild-type and mutant amino acids differ in size. The mutant residue is bigger, this might lead to bumps. |
| N209K | 9 | W<M | Neu →Pos |  | The mutant residue is bigger than the wild-type residue. The wild-type residue charge was NEUTRAL, the mutant residue charge is POSITIVE. There is a difference in charge between the wild-type and mutant amino acid. The mutation introduces a charge, this can cause repulsion of ligands or other residues with the same charge. The wild-type and mutant amino acids differ in size. The mutant residue is bigger, this might lead to bumps. |
| G210R |  | W<M | Neu →Pos |  | The mutant residue is bigger than the wild-type residue. The wild-type residue charge was NEUTRAL, the mutant residue charge is POSITIVE. The wild-type residue is more hydrophobic than the mutant residue. There is a difference in charge between the wild-type and mutant amino acid. The mutation introduces a charge, this can cause repulsion of ligands or other residues with the same charge. The wild-type and mutant amino acids differ in size. The mutant residue is bigger, this might lead to bumps. The torsion angles for this residue are unusual. Only glycine is flexible enough to make these torsion angles, mutation into another residue will force the local backbone into an incorrect conformation and will disturb the local structure. |
| G214R |  | W<M | Neu →Pos | ↑ | The mutant residue is bigger than the wild-type residue. The wild-type residue charge was NEUTRAL, the mutant residue charge is POSITIVE. The wild-type residue is more hydrophobic than the mutant residue. There is a difference in charge between the wild-type and mutant amino acid. The mutation introduces a charge, this can cause repulsion of ligands or other residues with the same charge. The wild-type and mutant amino acids differ in size. The mutant residue is bigger, this might lead to bumps. The torsion angles for this residue are unusual. only glycine is flexible enough to make these torsion angles, mutation into another residue will force the local backbone into an incorrect conformation and will disturb the local structure. |
| F218L |  | W>M |  |  | The mutant residue is smaller than the wild-type residue. The wild-type and mutant amino acids differ in size. The mutant residue is smaller, this might lead to loss of interactions. |
